# A spectrum of gut microbiota signatures predicts long-term health status

**DOI:** 10.1093/bioinformatics/btag484

**Published:** 2026-08-21

**Authors:** Himmi Lindgren, Chandler Ross, Matti Ruuskanen, Veikko Salomaa, Rob Knight, Teemu Niiranen, Aki Havulinna, Leo Lahti

**Affiliations:** Department of Computing, University of Turku, Turku FI-20014, Finland; Department of Computing, University of Turku, Turku FI-20014, Finland; Department of Life Technologies, University of Turku, Turku FI-20014, Finland; Department of Internal Medicine, University of Turku, Turku FI-20014, Finland; Department of Pediatrics, University of California San Diego, LA Jolla, CA 92093, United States; Department of Clinical Medicine, University of Turku, Turku FI-20014, Finland; Department of Computing, University of Turku, Turku FI-20014, Finland; Department of Computing, University of Turku, Turku FI-20014, Finland

## Abstract

**Motivation:**

The human gut microbiota is a complex ecosystem composed of hundreds of unique microbial species in a typical adult individual. Its composition has been linked to an individual’s current and future health. At the population level, joint analysis of co-abundant species can capture associations with health status and other variables that cannot be robustly detected by analyzing individual taxa alone. Recently, the concept of enterosignatures was proposed to detect such co-abundant groups, decomposing microbial community variation into a mixture of five broad components of co-varying microbial taxa. However, their overall robustness, generalizability, and prospective potential in predicting long-term health status remain to be evaluated.

**Results:**

We demonstrate that robust signatures of co-abundant species can be prospectively associated with mortality, diabetes, liver disease, and sepsis risk among Finnish adults in the FINRISK population cohort based on a 20-year follow-up. We characterized dysbiosis-associated signatures across varying levels of sub-ecosystem granularity, encompassing a previously reported *Escherichia*-driven signature as well as novel *Prevotella* subspecies-driven signatures linked to compromised future health status. These results illustrate the benefits of analyzing latent signatures across multiple levels of ecosystem granularity.

## 1 Introduction

**Figure 1:**
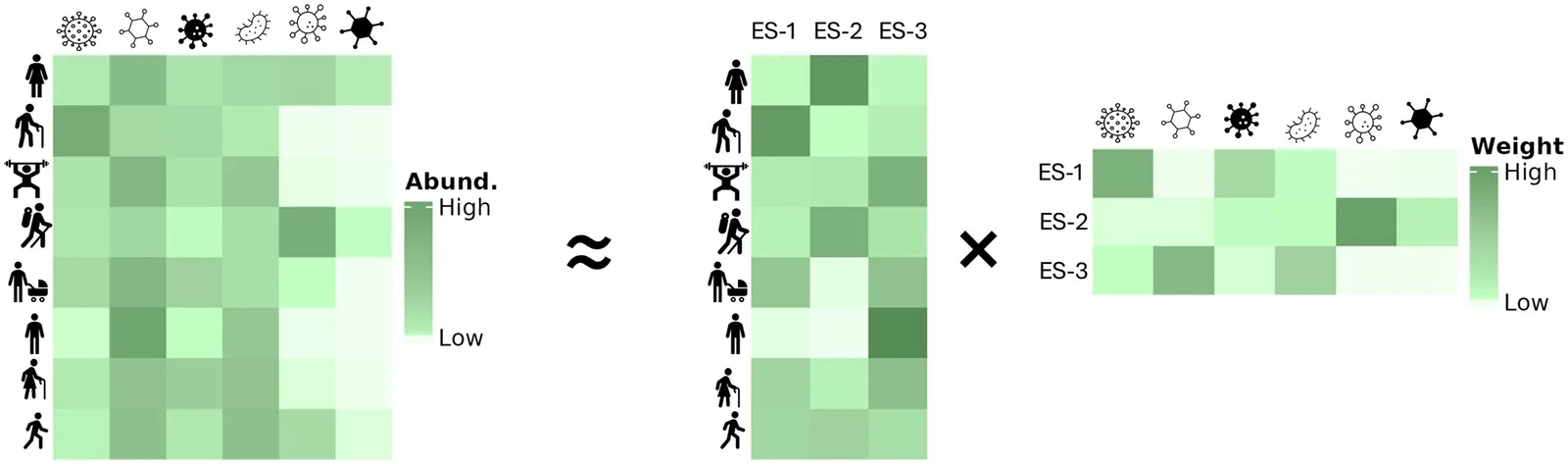
Overview of latent factorization into gut microbiota signatures. Schematic illustration of a latent factorization of fecal microbiota profiles into three sub-communities. The observed microbial abundance data table (left) is represented as a mixture of three latent signatures (*ES*). Each individual has a unique mixed membership of the signatures with varying weights (middle), and each signature corresponds to a specific community profile (right).

Ecological modeling aims to represent complex biological systems in an interpretable form. The high dimensionality of gut microbiota data, along with high variance and inter-individual diversity, poses challenges for statistical modeling (Jeganatha and Holmes 2021). Clustering (Arumugam *et al.* 2011) and latent mixture models (Holmes *et al.* 2012, Frioux *et al.* 2023) have been proposed to reduce the dimensionality of gut microbiota profiles by identifying co-abundant taxa that characterize sub-communities within the population. Depending on the method, each sample is either assigned to a single sub-community or can have a varying degree of membership across sub-communities. We refer to these sub-communities as gut microbiota signatures (Fig. 1).

Several approaches have been proposed for modeling of the gut ecosystem. Two main concepts are clustering into discrete community types called enterotypes (*ET*s) (Arumugam *et al.* 2011, Costea *et al.* 2018), where each sample is assigned to a single enterotype, and decomposing abundance matrices into continuous factors known as enterosignatures (*ES*s) (Frioux *et al.* 2023), where each sample has a degree of membership across all enterosignatures. Whereas the conceptualization of enterotypes remains controversial (Knights *et al.* 2014, Costea *et al.* 2018), many studies have described the main enterotypes driven by one of the three dominant genera: *Bacteroides*, *Prevotella*, and *Ruminococcus* of the phylum *Firmicutes* (Vandeputte *et al.* 2017, Costea *et al.* 2018, Aasmets *et al.* 2022). Subsequent research has extended this by allowing overlapping memberships across sub-communities (Knights *et al.* 2014, Lahti *et al.* 2014, Frioux *et al.* 2023). Enterosignatures allow for the identification of the co-abundance of community types in each sample. Frioux *et al.* (2023) distinguished between five enterosignatures at the genus level: one dominated by *Bacteroides*, one by *Prevotella*, one by *Bifidobacterium*, another by *Escherichia*, and the last by genera in the phylum *Firmicutes*. These were recently replicated in an independent cohort of Finnish adults (Pärnänen *et al.* 2025). Thus, mixed membership models provide promising tools for characterizing the human gut microbiota (Knights *et al.* 2014, Lahti *et al.* 2014, Hosoda *et al.* 2020, Xiong *et al.* 2023).

Modeling the human gut microbiota bridges ecology and biomedical research, as one of its aims is to characterize how the ecological structures of the human gut microbiota relate to present and future health outcomes. Several disease endpoints, such as cancer, liver disease, type 2 diabetes, and sepsis, have been associated with variation in the gut ecosystem (Wang *et al.* 2012, Ruuskanen *et al.* 2021, 2022, Pärnänen *et al.* 2025). The *Enterobacteriaceae* family has been associated with compromised health, and shown to predict increased long-term mortality risk (Salosensaari *et al.* 2021, Pärnänen *et al.* 2025). The *Firmicutes* family, on the contrary, has been linked to better health; several members of this family produce short-chain fatty acids, which regulate intestinal homeostasis and have anti-inflammatory properties (Fusco *et al.* 2023, Bah *et al.* 2025). Similar health associations have been reported with a *Firmicutes* enterosignature and a *Ruminococcus* enterotype (Costea *et al.* 2018, Frioux *et al.* 2023).

Broad community structures identified by latent mixture models have been associated with variations in health status. For instance, the *Bacteroides*-driven signature was recently identified and correlated with prior antibiotic intake (Frioux *et al.* 2023), similar to the *Bacteroides* driven enterotype (Aasmets *et al.* 2022). Moreover, it has also been associated with obesity and hypertension (Bah *et al.* 2025). Previous findings reported high levels of *Bacteroides* in samples with increased mortality risk (Wilmanski *et al.* 2021). In contrast, the *Prevotella*-driven signature was correlated with a lower intake of antibiotics and a fiber-rich diet, related to better health (Frioux *et al.* 2023). In addition, *Prevotella*-rich gut microbiota has been associated with enhanced weight loss and lowered cholesterol levels (Tett *et al.* 2021). Interestingly, the health-compromising roles of *Prevotella* have also been observed in mouse and human studies: *Prevotella copri* is one of the indicator species associated with insulin resistance, and in mice it has been shown to induce insulin resistance and glucose intolerance (Pedersen *et al.* 2016), as well as the *Prevotella* enterotype has been associated with an increased risk of obesity and diabetes mellitus (Bah *et al.* 2025).

Microbes are classified using taxonomy-based classifications. The taxonomic levels, such as species, genera, and families, aggregate microbial diversity into hierarchical taxonomic groupings. Microbial communities consist of interacting and co-abundant groups of taxa, which can reflect relationships extending beyond taxonomic groupings. Microbial co-abundance and community types have been commonly modeled with clustering techniques, which implicitly assume mutually exclusive, discrete groups. This may not fully reflect the complexity and dynamic nature of the gut ecosystem. To better reflect the ecosystem variation, we used continuous mixture models to characterize microbiota as a combination of co-occurring community types. We used latent factorization as described below, and increased the taxonomic resolution from genus (Frioux *et al.* 2023, Pärnänen *et al.* 2025) to species level. We then modeled the ecological landscape using up to 20 latent species-level signatures at increasing levels of granularity. We observed evolving combinations of co-abundant species. Furthermore, we associated these ecological structures with long-term health status and mortality in a prospective population-based cohort study with a 20-year follow-up.

## 2 Materials and methods

### 2.1 Study sample

FINRISK is a comprehensive Finnish population survey on risk factors for chronic diseases among Finnish adults (Borodulin *et al.* 2018). The surveys were conducted by the Finnish Institute for Health and Welfare (THL) in five-year intervals between 1972 and 2012. The surveys were based on stratified random samples of the Finnish adult population aged 25–74. Fecal samples were collected during the FINRISK 2002 survey (*n* = 7048), which represented most of the microbiome data, and again in a subsample of an independent cohort, the FINRISK 2007 survey (*n *= 301).

The study protocols for the FINRISK 2002 and 2007 surveys were approved by the Coordinating Ethical Committee of the Helsinki and Uusimaa Hospital District (Ref. 558/E3/2001, 229/E0/2006). The study was conducted in accordance with the World Medical Association’s Declaration of Helsinki on ethical principles. All participants signed an informed consent form.

#### 2.1.1 Microbiome sequencing from stool samples

Participants willing to provide stool samples received a kit with detailed instructions. The samples collected in 2002 were stored at $-20^{{}^{\circ}}C$ and in 2007 at $-70^{{}^{\circ}}C$ in the Finnish Institute for Health and Welfare laboratory. In 2017, they were transferred to the University of California, San Diego, for shallow shotgun metagenomic sequencing with untargeted accuracy against mapped reference databases. Host reads were removed by mapping them to the human genome assembly GRCh38 using Bowtie2. The filtered reads were mapped to the Greengenes2 (GG2) reference database (McDonald *et al.* 2024). The mapped reads were processed by the Woltka pipeline. Although recent taxonomic revisions propose new naming, for example, phylum *Firmicutes* as *Bacillota* and genus *Prevotella* as *Segatella*, we retain the names directly provided by the preprocessing pipeline.

#### 2.1.2 Register linkages

In Finland, each permanent resident has a unique personal identity code given at birth or after immigration, which ensures a reliable link to the health registers. National health registers are validated sources for all major health events (Care Register for Health Care), all deaths (Causes-of-death Register), and all medications prescribed by a doctor (Drug Reimbursement Register) (Gissler and Haukka 2004, Sund 2012). For this work, data were collected from health registers until December 2022. During this period, we observed 1160 deaths (all-cause) among 7141 participants. For common morbidity endpoints, we selected diabetes mellitus, liver disease, and sepsis related to bacterial infection, with 989, 151, and 242 incident events, respectively. We selected these endpoints due to their previously reported associations with gut microbiota (Ruuskanen *et al.* 2021, 2022, Salosensaari *et al.* 2021, Pärnänen *et al.* 2025). We evaluated the potential of latent signatures to enhance the detection of ecosystem structures that associate with future health.

### 2.2 Data analysis

The downstream data analysis was performed using R version 4.5.2. Data were stored in a TreeSummarizedExperiment object (Huang *et al.* 2021) and analyzed within the Bioconductor microbiome framework (Borman *et al.* 2025). We performed taxonomic feature filtering using 0.01% detection and 10% prevalence thresholds. After filtering, 467 species remained for further analyses. Samples with total read counts below 50 000 were filtered out, yielding 7141 samples.

#### 2.2.1 Latent factorization

The latent signatures represent the overall variation of gut microbiota abundances as a mixture of a few coherent components (Fig. 1). We obtained the latent signatures using non-negative matrix factorization (NMF) as in (Frioux *et al.* 2023), implemented in the R package *NMF* (version 0.28), applied to an untransformed count-per-sample matrix, with 8 runs each. This preserved information of the sequencing depth.

NMF minimizes the Kullback-Leibler divergence between the data and its lower rank approximation $\min D_{KL}(V||WH)$, where *V* $\in R_{\geq 0}^{n\times m}$ is the original data matrix, and the matrix product of *W* $\in R_{\geq 0}^{n\times k}$ and *H* $\in R_{\geq 0}^{k\times m}$ approximates *V* for each *k* ∈ {1,2,…,20} (Maisog *et al.* 2021).

As part of the model selection, we compared different values of *k* based on cosine similarity (Fig. S1, available as supplementary data at *Bioinformatics* online). Similarity increased with *k*, showing a local maximum at *k *= 16 and reaching its highest value over the explored range at *k *= 20. Overall, reconstruction quality improved across the tested range, with diminishing marginal gains at higher values of *k*. We therefore selected *k *= 20 as a high-granularity model, prioritizing reconstruction quality. This was compared against a lower-complexity standard model with *k *= 5, which corresponds to the originally published number of signatures (Frioux *et al.* 2023).

We qualitatively named the latent signatures using their principal indicator species from the *H* matrix and the similarity between standard and high-granularity models, obtained via component alignment. In the standard model, we shortened the names, and in the high-granularity model, we used the name of the indicator species.

#### 2.2.2 Component alignment

Next, we investigated how low-granularity signatures split into increasingly high-resolution signatures with increasing values of *k*. We performed component alignment for sample matrices using the *alto* R package (version 0.1.0) as proposed in (Fukuyama *et al.* 2023). The component alignment framework is based on a weighted graph in which each node represents a component of one of the 20 models (*k* ∈ {1,2,.,20}), and the edges quantify the similarity between the components in the previous and subsequent models $W_{k}^{⊤}W_{k+1}$.

#### 2.2.3 Cross-sectional correlation analysis

First, we evaluated how the latent signatures cross-sectionally associate with host attributes. We focused on the standard granularity of *k *= 5 (Frioux *et al.* 2023). We applied pairwise Kendall’s tau correlation coefficient to assess associations between the *ES* membership and participant age, sex, body-mass index (BMI), antibiotic use (prescription, defined as ATC code of J01 up to six months before baseline; *n *= 1269), pregnancy (*n *= 42), and vegetarian diet (self-reported, *n *= 225). In addition, we analyzed associations with the selected morbidities: prevalent diabetes (*n *= 307), previous liver disease (*n *= 53), and prior sepsis (*n *= 55). We considered the baseline associations with *P *< 0.05 as potential confounders in the prospective analysis.

#### 2.2.4 Prospective survival analysis

We investigated time-to-event associations of the latent signatures with morbidity and mortality endpoints. First, we investigated prospective associations with mortality using all *k* levels of granularity (*k* ∈ {1,2,.,20}). We used *k* model components as multivariate predictors in Cox proportional-hazards using the R package *survival* (version 3.8–3). For mortality endpoints, we therefore fitted 20 Cox regression models altogether as $M_{k}$=Cox(Surv(time,death)  ∼  $X_{1}$+$X_{2}$+…+$X_{k})$. For the other endpoints, we used the standard model with *k *= 5 (Frioux *et al.* 2023, Pärnänen *et al.* 2025), and additionally *k *= 20 as a high-granularity model. This allowed us to compare alternative approaches: a low-granularity representation, which can be more straightforward to interpret, and a high-granularity representation, which captures more nuanced structures in the data. Then, we ran multivariate Cox regressions for disease endpoints using the continuous signatures as predictors, $M_{\mathrm{event},k}$ = Cox(Surv(time, event) $∼X_{1}+X_{2}+\ldots +X_{k}$), where *k* ∈ {5, 20}.

In continuous analyses, hazard ratios (HR) are reported per $10^{6}$-unit increase in the predictor. We controlled multiple hypothesis testing on all 285 predictor effects tested ($k_{\mathrm{death}}$ ∈ {1,2,.,20}, $k_{\mathrm{diab}}$ ∈ {5,20}, $k_{\mathrm{liver}}$ ∈ {5,20}, $k_{\mathrm{sepsis}}$ ∈ {5,20}) by adjusting the *P-*value using the Benjamini–Hochberg FDR procedure. We considered *q *< 0.05 to be statistically significant.

In discrete analyses, we set a discrete maximum membership, representing the principal *ES* for each sample. $M_{c}$=Cox(Surv(time,  death)  ∼  $ES_{c})$, where *c* ∈ {5, 20}. Here, we analyzed only the mortality endpoint and used *ES-Bact. H uniformis* as the reference. We controlled multiple testing separately for each model.

#### 2.2.5 Supporting analyses

Finally, we performed two supporting analyses: first, to evaluate the predictive performance of the signatures relative to individual genera, and then as a sensitivity analysis to assess batch effects and potential confounding. For the first analysis, we compared signatures obtained from the granularity level *k *= 20 with the main genera represented by these signatures. Both were inverse-rank-transformed to facilitate comparison between *ES* and genus abundances. We ran separate univariate Cox regression for each of these univariate predictors with respect to total mortality (Table S5; Fig. S2, available as supplementary data at *Bioinformatics* online). Then, for the second analysis, we analyzed mortality associations only in the FINRISK 2002 cohort using *k *= 5 and *k *= 20 signatures, and included age, sex, BMI, and antibiotic use as covariates in the additional analysis (Table S6; Fig. S3, available as supplementary data at *Bioinformatics* online).

## 3 Results

### 3.1 Cross-sectional associations

**Figure 2:**
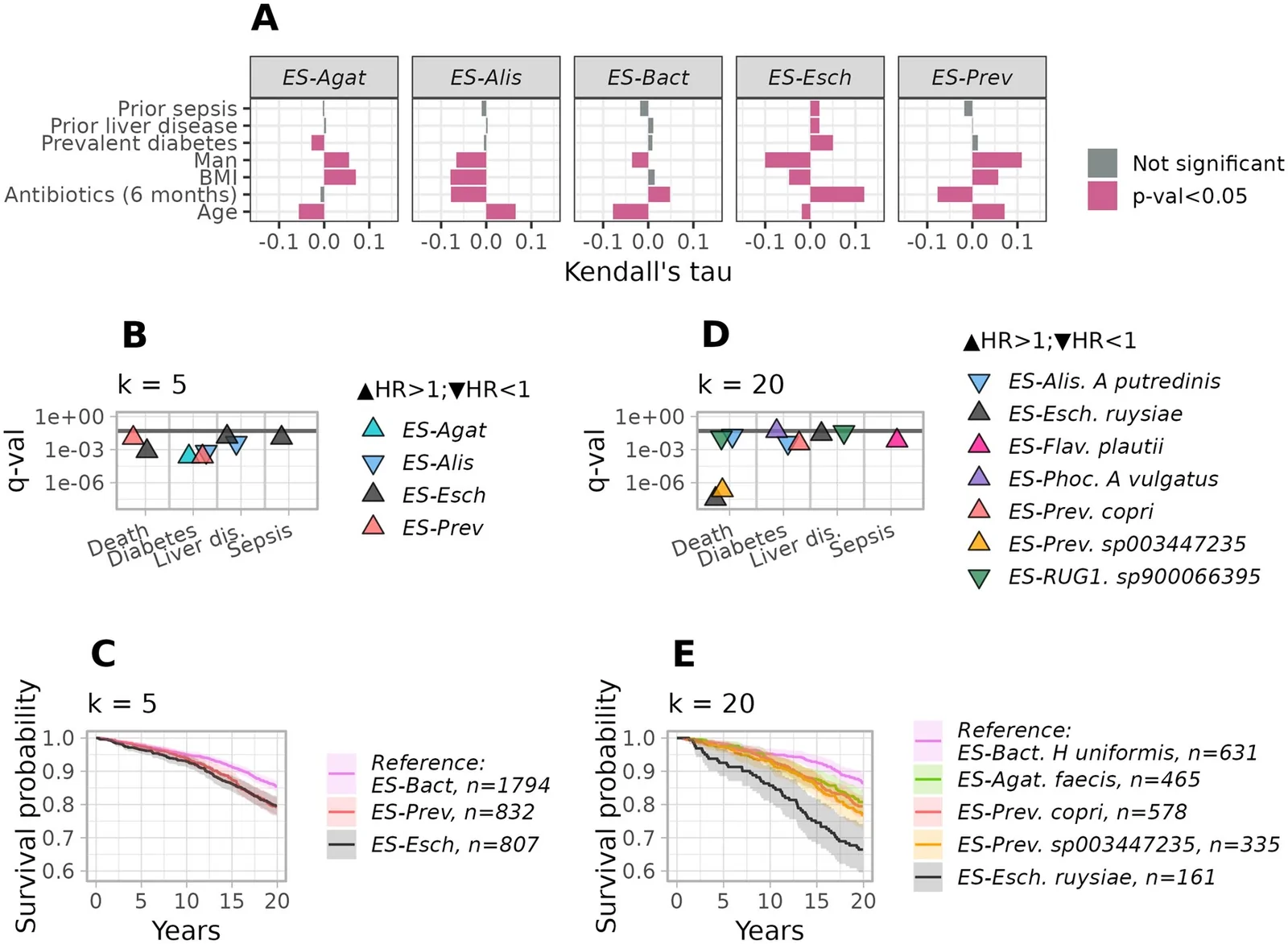
Both standard and high-granularity signatures were associated with mortality and common morbidities. (A) Pairwise cross-sectional correlation of the signatures (*k* = 5) with significant health variables. (B) *k* = 5 granularity level. Four multivariate Cox regression models for all-cause mortality, incident diabetes, liver disease, and sepsis responses. An upward-pointing triangle represents HR > 1, and a downward-pointing triangle represents HR < 1. (C) *k* = 5 granularity level. Kaplan-Meier (KM) analysis for categorized all-cause mortality response, where *ES-Bact* is set as the reference level. Only significant (*q*  < 0.05) principal *ES*s are plotted. (D) *k* = 20 granularity level. Four multivariate Cox regression models for all-cause mortality, diabetes, liver disease, and sepsis responses. (E) *k* = 20 granularity level. KM analysis for categorized all-cause mortality response, where *ES-Bact. H uniformis* is set as the reference level. Only significant principal *ES*s are plotted.

We examined retrospective and cross-sectional data as a starting point for the analyses described previously. The cross-sectional correlations are summarized in Fig. 2A (Table S2, available as supplementary data at *Bioinformatics* online) and represent the baseline characteristics of the samples that describe these signatures. Aligned with previous studies, we described *ES-Prev* to be positively correlated with higher BMI (τ = 0.06; *P* = 5.7e-13) (Bah *et al.* 2025), and negatively correlated with antibiotic use (τ = −0.08; *P* = 2.3e-15) (Frioux *et al.* 2023), as well as both *ES-Bact* and *ES-Esch* to be positively correlated with antibiotic use (τ = [0.05,0.12]; both *P *< 1e-6) (Frioux *et al.* 2023). *ES-Esch* was also positively correlated with a history of sepsis (τ = 0.02; *P* = 0.037), previous liver disease (τ = 0.02; *P* = 0.043), prevalent diabetes (τ = 0.05; *P* = 2.7e-7), and lower BMI (τ = −0.05; *P* = 3.4e-9), suggesting that individuals with a high membership of *ES-Esch* may already have been less healthy at the beginning of follow-up. Instead, *ES-Alist* was correlated with less antibiotic use (τ = −0.08; *P* = 6.9e-16) and lower BMI (τ = −0.08; *P* < 1e-16), and *ES-Agat* with no prevalent diabetes (τ = −0.03; *P* = 0.0034), and higher BMI (τ = 0.07, *P* < 1e-16). Moreover, all signatures correlated with sex and age; *ES-Agat* and *ES-Prev* were correlated with male sex (τ = [0.06,0.11]; both *P* < 1e-7) and *ES-Bact*, *ES-Alis*, and *ES-Esch* with female (τ = [−0.04,−0.07,−0.10]; all *P* < 1e-3), while *ES-Alis* and *ES-Prev* were positively correlated with older age (τ = [0.07,0.07]; both *P* < 1e-16), and *ES-Agat*, *ES-Bact*, and *ES-Esch* with younger (τ = [−0.06,−0.08,−0.02]; all *P* < 0.05). We did not detect association between diet and pregnancy status for any of the signatures.

### 3.2 Prospective associations

**Figure 3:**
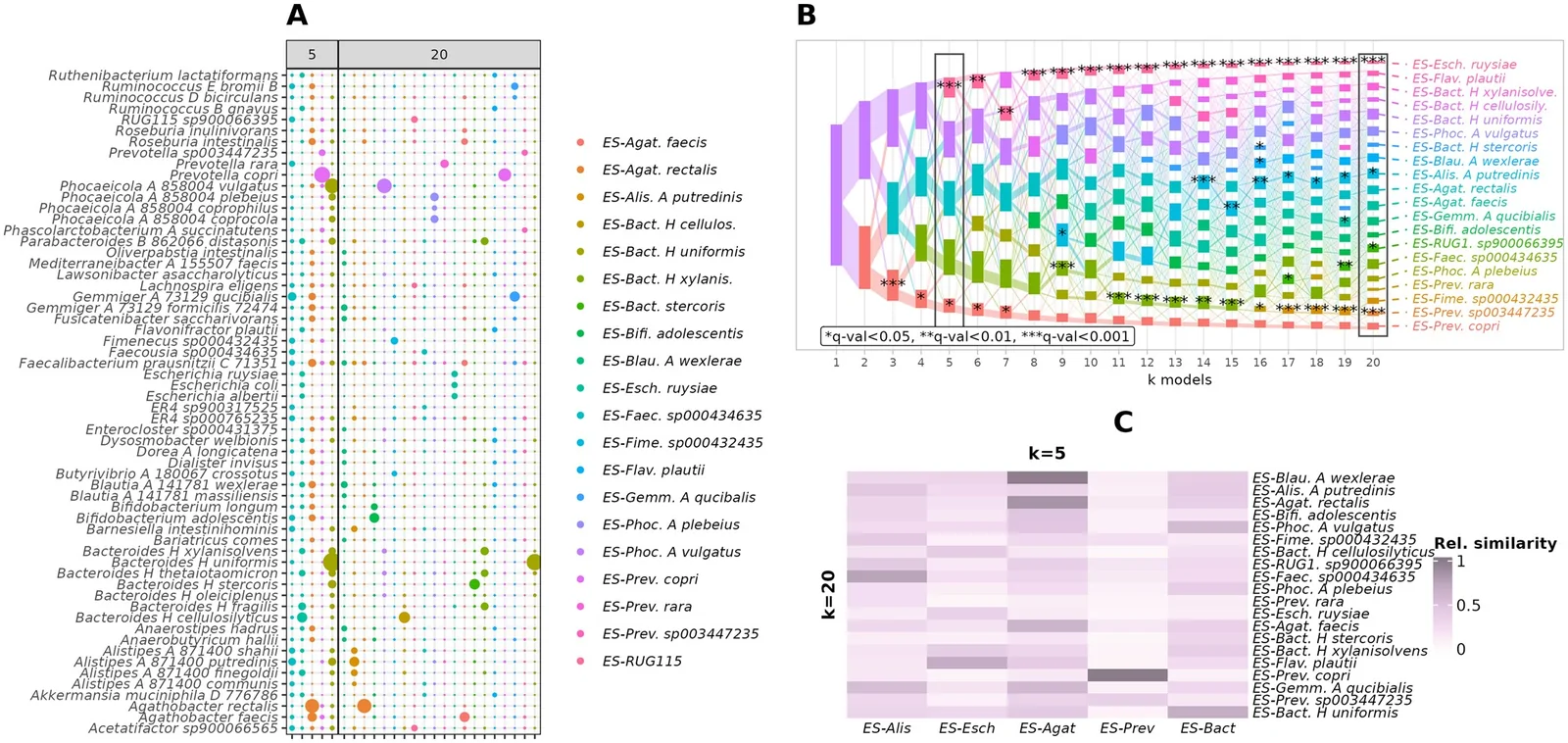
Granularity-robust similarity captures the features of the mortality-associated paths across the alignment. (A) The relative composition of each of the 60 unique indicator species combined both in the standard (*k* = 5) and high-granularity (*k* = 20) models. Circle size refers to the magnitude of each species’ contribution to the *ES*. (B) Structure of component alignment across 20 models (*k* ∈ {1,2,…,20}), where the nodes represent each *ES* in each model, and the width of the edges represents the similarity (inner product) of the signatures between *k* and *k*+1. Illustrates how the signatures evolve with *k*. Statistically significant results from multivariate Cox regression models for all-cause mortality response. (C) Relative similarity between the standard (*k* = 5) and high-granularity (*k* = 20) models, representing the divergence of the signatures. Emphasizes that eubiotic *ES-Alis* (*k* = 5) is a parent of many signatures including *ES-Alis. A putredinis* and *ES-RUG115*, as well as dysbiotic *ES-Esch*, is a parent signature of *ES-Esch. ryusiae* and *ES-Flav. plautii*, and *ES-Prev* primarily of *ES-Prev. copri*.

We analyzed community structure at different levels of granularity, identifying signatures associated with future mortality and morbidity risk (Figs. 2B–E, 3B). We demonstrated the observations using both continuous (Table S1, available as supplementary data at *Bioinformatics* online) and categorized signatures (Tables S3 and S4, available as supplementary data at *Bioinformatics* online), as described previously.

#### 3.2.1 Standard model

*ES-Prev* was associated with higher mortality (HR, 1.03; 95%CI, 1.01–1.05; *q *= 0.011) and incident diabetes risk (HR, 1.04; 95%CI, 1.02–1.06; *q *= 2.3e-4), whereas *ES-Alis* with lower risk for both diabetes (HR, 0.92; 95%CI, 0.88–0.96; *q *= 6.4e-4) and liver disease (HR, 0.73; 95%CI, 0.61–0.87; *q *= 0.0042) (Fig. 2B). *ES-Agat* was associated with an increased risk of diabetes (HR, 1.05; 95%CI, 1.02–1.07; *q *= 2.3e-4), although this effect did not persist in the high-granularity model. Furthermore, *ES-Esch* was associated with a higher risk of sepsis (HR, 1.06; 95% CI, 1.02–1.11; *q *= 0.012) in addition to the higher risk of mortality (HR, 1.04; 95%CI, 1.02–1.06; *q *= 7.2e-4) and liver disease (HR, 1.07; 95%CI, 1.02–1.11; *q *= 0.015). This association of sepsis was captured by *ES-Flav. plautii*, a child-signature of *ES-Esch*, in the high-granularity model.

The categorized Cox regression model detected *ES-Esch* (HR, 1.47; 95%CI, 1.21–1.79; *q *= 2.5e-4) and *ES-Prev* (HR, 1.48; 95%CI, 1.21–1.79; *q *= 2.5e-4) signatures with significant association with higher mortality risk using *ES-Bact* as a reference level (Fig. 2C).

#### 3.2.2 High-granularity model

In the high-granularity model (Fig. 2D), the *ES-Prev* signature had divided into two *Prevotella* sub-signatures, of which *ES-Prevotella copri* was associated with an elevated risk of diabetes (HR, 1.02; 95%CI, 1.01–1.04; *q *= 0.0036), and *ES-Prevotella sp003447235* with higher mortality risk (HR, 1.04; 95%CI, 1.03–1.06; *q *= 2.0e-7). The eubiotic signatures *ES-Alistipes A putredinis*, *ES-RUG115 sp00066395* were positively associated with survival: *ES-RUG115* in mortality (HR, 0.94; 95%CI, 0.91–0.98; *q *= 0.013) and liver disease (HR, 0.79; 95%CI, 0.66–0.94; *q *= 0.04), and *ES-Alis. A putredinis* in mortality (HR, 0.94; 95%CI, 0.90–0.98; *q *= 0.019) and diabetes (HR, 0.93; 95%CI, 0.89–0.97; *q *= 0.0042). In this high-granularity model, the separate signatures were associated with mortality and sepsis, *ES-Escherichia ryusiae* with a higher mortality risk (HR, 1.04; 95%CI, 1.03–1.05; *q *= 3.3e-8) and *ES-Flavonifractor plautii* with a higher risk of sepsis (HR, 1.04; 95%CI, 1.02–1.06; *q *= 0.0065). Moreover, we detected that *ES-Phocaiecola vulgatus* was associated with a higher risk of diabetes (HR, 1.03; 95%CI, 1.01–1.06; *q *= 0.048). Furthermore, compared to the univariate high-granularity signatures of their top indicator genera (Table S5; Fig. S2, available as supplementary data at *Bioinformatics* online), *ES-Prev. sp003447235* (HR, 1.25; 95%CI, 1.18–1.32; *q *= 4.4e-13) and *ES-Blau. A wexlerae* (HR, 0.78; 95%CI, 0.74–0.83; *q *= 1.9e-14) captured stronger associations with mortality compared to *Prevotella* (HR, 1.07; 95%CI, 1.01–1.13; *q *= 0.024) and *Blautia A* (HR, 0.89; 95%CI, 0.84–0.94; *q *= 1.2e-4) genera, suggesting that some signatures may capture mortality-related co-abundance patterns beyond genus-level groupings, although the most informative sub-ecosystem representation depends on the specific comparison.

The categorized Cox regression model was generally consistent with the continuous results (Fig. 2E). The principal *ES*s significantly associated with mortality were *ES-Esch. ryusiae* (HR, 2.96; 95%CI, 2.10–4.18; *q *= 1.1e-8) and *ES-Prev. sp003447235* (HR, 1.83; 95%CI, 1.34–2.50; *q *= 0.0015), aligned with the previous results. Additionally, in this model, also *ES-Prev. copri* (HR, 1.62; 95%CI, 1.22–2.16; *q *= 0.0049) and *ES-Agat. faecis* (HR, 1.51; 95%CI, 1.12–2.04; *q *= 0.034), the latter of which did not reach significance in the continuous model, were also associated with higher mortality risk compared to *ES-Bact. H uniformis*.

#### 3.2.3 Paths of signatures

Inspired by Fukuyama *et al.* (2023), we aligned each signature to its most similar counterpart in the adjacent model. The signature structure remained largely consistent across granularity levels, as shown in Fig. 3B, supporting multiple valid choices of *k*, from the original five-component model to higher-granularity solutions. Survival models at granularity levels ranging from *k *= 3 to *k *= 20 identified multiple associations with the mortality endpoint. *ES-Alist. A putredinis* and *ES-RUG115* conveyed granularity-robust eubiotic effects, whereas *ES-Esch. ryusiae* and *ES-Prev. sp003447235* dysbiotic.

*ES-Esch* was the most consistent and distinct path and was associated with a higher risk of mortality from *k *= 5 to *k *= 20, with higher granularity revealing more informative signatures. The *ES-Prev* path exhibited a different pattern. At lower granularity from *k *= 3 to *k *= 7, *ES-Prev* was associated with an increased risk of mortality. From *k *= 8 onward, the *ES-Prev* path persisted, but the mortality association was lost. Instead, a new path driven by *Fimenecus sp000432435*, *Faecusia sp000434635*, and *Prevotella sp003447235* emerged and captured a mortality association at *k* ∈ {9,11–15}. At *k *= 16 *Faecusia sp000434635* and *Fimenecus sp000432435* driven paths, of which *ES-Fime. sp000432435* captured the mortality association, only until *k *= 17, when *Prev. sp003447235* driven path diverged, capturing the persistent mortality association. This transition suggests that *Prevotella* sub-species, and latent signatures driven by them, may have potential value for prospective health prediction. Furthermore, *ES-Alis* captured the most significant and robust eubiotic path at *k *= 9 and *k *= 14 onward. Other signatures associated with survival were *ES-RUG115* at *k* ∈ {17,19,20} and *ES-Blautia A wexlerae* at *k *= 16.

### 3.3 Granularity-robust similarity

In this study, we detected two dysbiotic paths associated with higher risks of mortality and morbidity endpoints, one driven by species of the family *Enterobacteriaceae* and the other by taxa of the genus *Prevotella*. The evaluated similarity is represented in Fig. 3C, which captures the parent-child similarities of the signatures between standard and high-granularity models. We observed that the *ES-Esch* signature (*k *= 5) is a parent of several signatures, the most important of which are *ES-Bact. H cellulosilyticus*, *ES-Bact. H xylanisolvens*, *ES-Esch. ruysiae*, *ES-Flav. plautii*. Similarly, *ES-Alis* and *ES-Bact* were observed to be parents of several high-granularity signatures. *ES-Agat* was observed to be very similar to *ES-Agathobacter rectalis* and *ES-Blautia A wexlerae*. However, it was, in general, the most similar standard signature to the high-granularity signatures. *ES-Prev* at *k *= 5, instead, was the most similar only to the *ES-Prev. copri* and *ES-Prev. sp003447235* signatures. It appears that in the high-granularity model, *ES-Prev. copri* represents the ultimate of *ES-Prev*, whereas *ES-Prev. sp003447235* is a mixture of *ES-Prev* and other signatures.

Finally, we characterized the feature landscapes of the standard and high-granularity models, as shown in Fig. 3A. We observed feature patterns for signatures detected by both standard and high-granularity models. When comparing standard and high-granularity models, we observed that the feature vectors began to converge on specific taxa at *k *= 20, whereas at *k *= 5 they were distributed across many taxa. In the high-granularity model, for instance *ES-Agat. rectalis*, *ES-Bact. uniformis*, and *ES-Prev. copri* were primarily driven by a single feature. These differences reflect that some sub-communities can be defined by lower-resolution representations or even by the abundance of a single taxon, while other co-abundance patterns require more nuanced factorization.

## 4 Discussion

This study contributes to the ongoing discussion that describes the gut ecosystem as a continuous mixture of signatures (Lahti *et al.* 2014, Frioux *et al.* 2023). In particular, this work considers these signatures as a spectrum at varying levels of granularity (Sankaran and Holmes 2019, Fukuyama *et al.* 2023). These approaches differ from clustering-based community typing, such as the Dirichlet Multinomial Mixtures (Holmes *et al.* 2012), which assume that each sample belongs to a single community type. Extending to continuous mixtures, NMF (Frioux *et al.* 2023) and Latent Dirichlet Allocation (Sankaran and Holmes 2019, Hosoda *et al.* 2020) have been used to identify such latent mixed memberships, both modeling the community as a mixture of non-negative components of co-abundant species detected in the data. Clustering can be viewed as a special case of a mixture with binary weights, analogous to using principal *ES*s. We demonstrated that both continuous signatures and discrete principal *ES*s can capture gut microbiota signatures associated with elevated mortality risk, yet they reflect different conceptualizations of ecosystem structure. The distinction between them becomes particularly relevant for intermediate samples that are not dominated by a single principal *ES*.

The latent mixture models are unsupervised, as the community structures are identified without using information on health or other external variables. Whereas the identified components are inherently agnostic to biomedical relevance, they are ecologically coherent and can be contrasted to the available parameters of host health. This can highlight naturally occurring structures within the ecosystem. The increasing granularity of the model can be seen as a way to discover substructures and refine broader signatures. The level of granularity, or the number of components (*k*), is a deliberate analytical decision that determines the resolution and depends on the research objective. Future research could aim to define simplified proxies for the signatures, such as direct sums of species abundances, and explore ways to enhance cross-cohort replicability based on these.

Selecting the level of granularity within context may help identify co-abundant species that reflect coherent ecosystem structures linked to specific health variation. For instance, we observed that the *Flav. plautii*-driven signature diverges from the *Escherichia*-driven signature at a higher level of granularity and is associated with a higher risk of sepsis. This is consistent with the systematic evidence that *Flav. plautii* is a potential pathogen for sepsis (Kvernberg *et al.* 2026). In addition, *Flavonifractor*, and especially *Escherichia*, genera may carry higher antimicrobial resistance gene loads, which may contribute to their associations with adverse outcomes (Pärnänen *et al.* 2025). The *Escherichia*-driven signature was also associated with an elevated risk of liver disease. Eubiotic associations, in contrast, were reflected in signatures driven by *Alistipes* and *RUG115*. Specifically, the path driven by *Alistipes A 871400 putredinis* was associated with lower mortality risk and reduced diabetes risk, supported by previous evidence (Ruuskanen *et al.* 2022). The genus *Prevotella* is another interesting example, comprising a highly diverse group of species with distinct metabolic capabilities (Yeoh *et al.* 2022), which aligns with our findings of divergence in *Prevotella* subspecies-driven paths associated with higher mortality risk. At lower levels of granularity, the *Prevotella*-driven path appeared as a single signature, but it diverged into multiple sub-signatures at higher levels of granularity. Among these, the *Prev. copri*-driven signature was specifically associated with a higher risk of diabetes, aligned with previous research (Bah *et al.* 2025), and the novel *Prev. sp003447235*-driven signature with an elevated mortality risk.

The gut microbiota is a dynamic ecosystem that comprises a rich mixture of ecosystem types, observed at variable population frequencies (Lahti *et al.* 2014, Frioux *et al.* 2023, Fukuyama *et al.* 2023). The cross-sectional taxonomic data represent a single point in time for the Finnish population, which limits this work, as we cannot evaluate the stability of the observed signatures over time. The emerging signatures driven by *Prev. sp003447235* and *Flav. plautii*, detected only in high-granularity models, represent less common configurations of the ecosystem. This might reflect a temporal transition stage captured at a single time point or distinct but rare ecosystem types. Treating them as separate signatures from *Prev. copri* and *Escherichia*-driven signatures revealed clinical evidence supporting the interpretation of unique sub-ecosystems rather than merely transitional stages. Further longitudinal data collection can help to further elucidate this and other dynamical aspects.

In summary, latent factorization models can uncover a spectrum of co-abundant species at varying levels of granularity. We detected ecologically coherent signatures linked to altered morbidity and mortality risk in a long-term follow-up. This contributes to the ongoing discussion on modeling the human gut microbiota, capturing species-level co-abundance and prospective associations.

## 5 Conclusion

This study demonstrates the advantages of latent factorization models, in particular the NMF, for modeling the structure of the gut ecosystem across multiple levels of community granularity in population-scale taxonomic profiling data. We show that increasing sub-community granularity helps to stratify high-risk samples and identify subpopulations at elevated risk of early mortality. Our analyses also advanced our understanding about gut signatures as observational risk markers for disease incidence. We identified significant prospective associations over a 20-year follow-up period with incident type 2 diabetes, liver disease, and sepsis. These observations highlight the potential of latent factorization for detecting clinically relevant species consortia in the human microbiota and broaden the spectrum of microbiota states associated with current and future health.

## Supplementary Material

btag484_Supplementary_Data

## Data Availability

The metagenomic data are available from the European Genome-Phenome Archive (accession number EGAD00001007035). The phenotype data contain sensitive information, and they are available through the THL biobank upon submission of a research plan and signing a data transfer agreement (https://thl.fi/en/our-services/thl-biobank1/how-to-apply-for-thl-biobank-data-and-samples). The source code for the analysis is available (https://doi.org/10.5281/zenodo.20920412).
