# Supplementary material for: A spectrum of gut microbiota signatures predicts long-term health status
Source: Bioinformatics. 2026 Aug 21;42(Suppl 2):btag484. doi: 10.1093/bioinformatics/btag484 (PMC13501278; doi:10.1093/bioinformatics/btag484)
Supplement: Supplementary file 1 [file btag484_supplementary_data.pdf]

Supplementary material

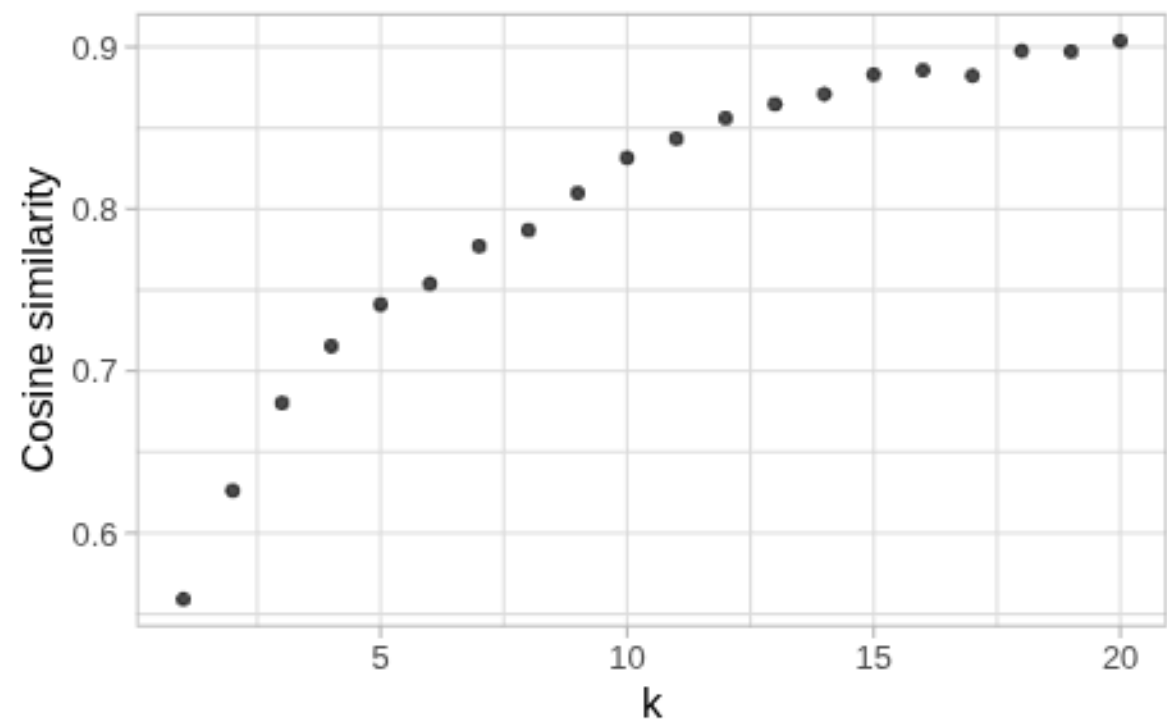

Figure S1. Cosine similarity represents the reconstruction quality between the original data and the lower dimensionality representation across k models. The similarity increases steadily, finding a local maximum at k=16. The similarity starts to plateau after k=18, however, still increasing with a moderately reduced marginal gain at k=19 to k=20. This supporting the selection of k=20 as a high-granularity model as it maximizes explained information.

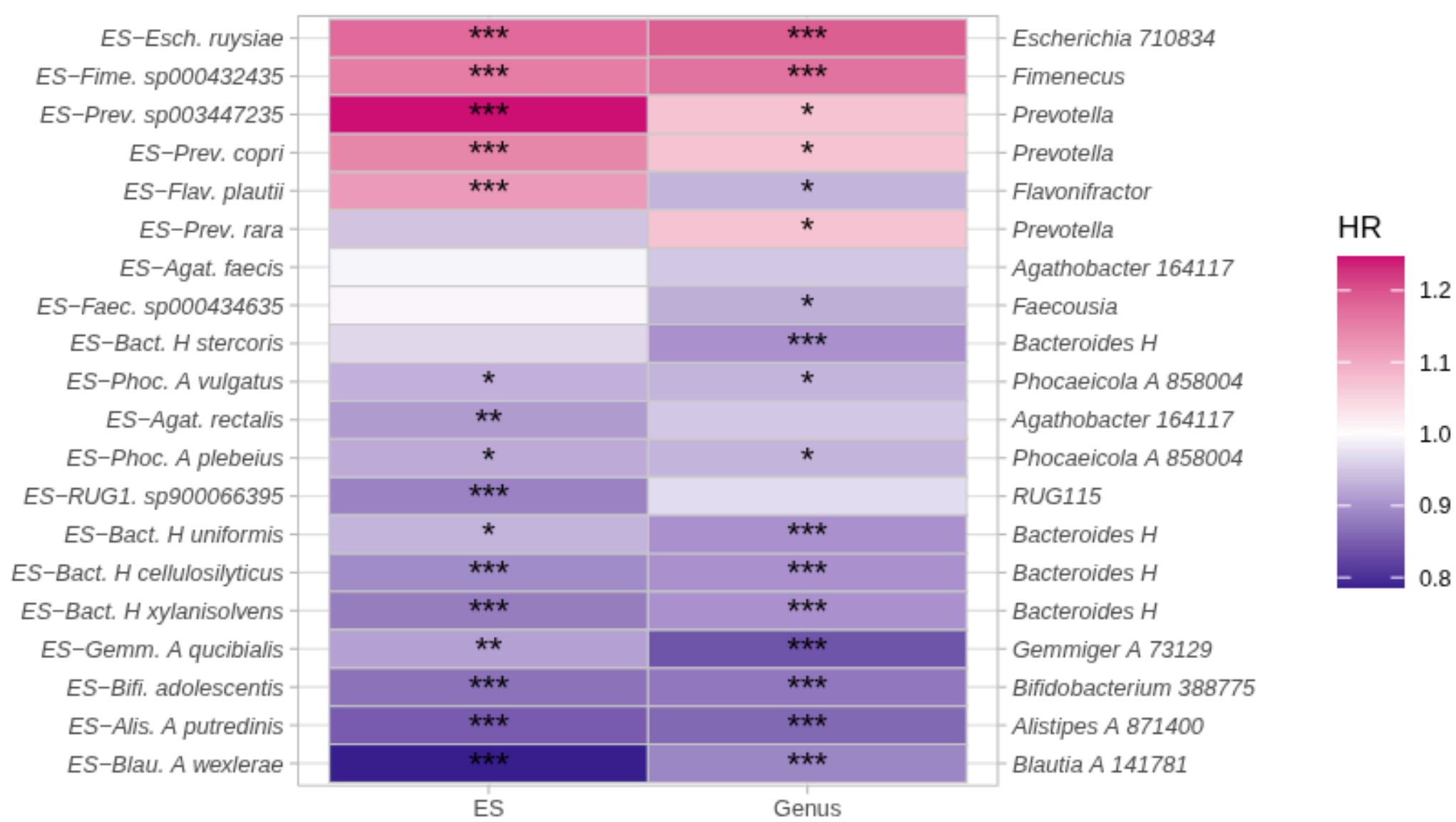

Figure S2. Cox proportional hazards regression (mortality) (* q<0.05; ** q<0.01; ***q<0.001). Here the hazard ratio of each *ES* is compared with the hazard ratio of the genus of the highest indicator species of the *ES*. Both signatures and genus abundances are inverse-rank-transformed for comparability. In general, the performance of the signatures is well equivalent with the performance of the representative genera, although *ES-Prev. sp003447235* and *ES-Blau. A wexlerae* has stronger association with mortality than *Prevotella* and *Blautia*, respectively, genera.

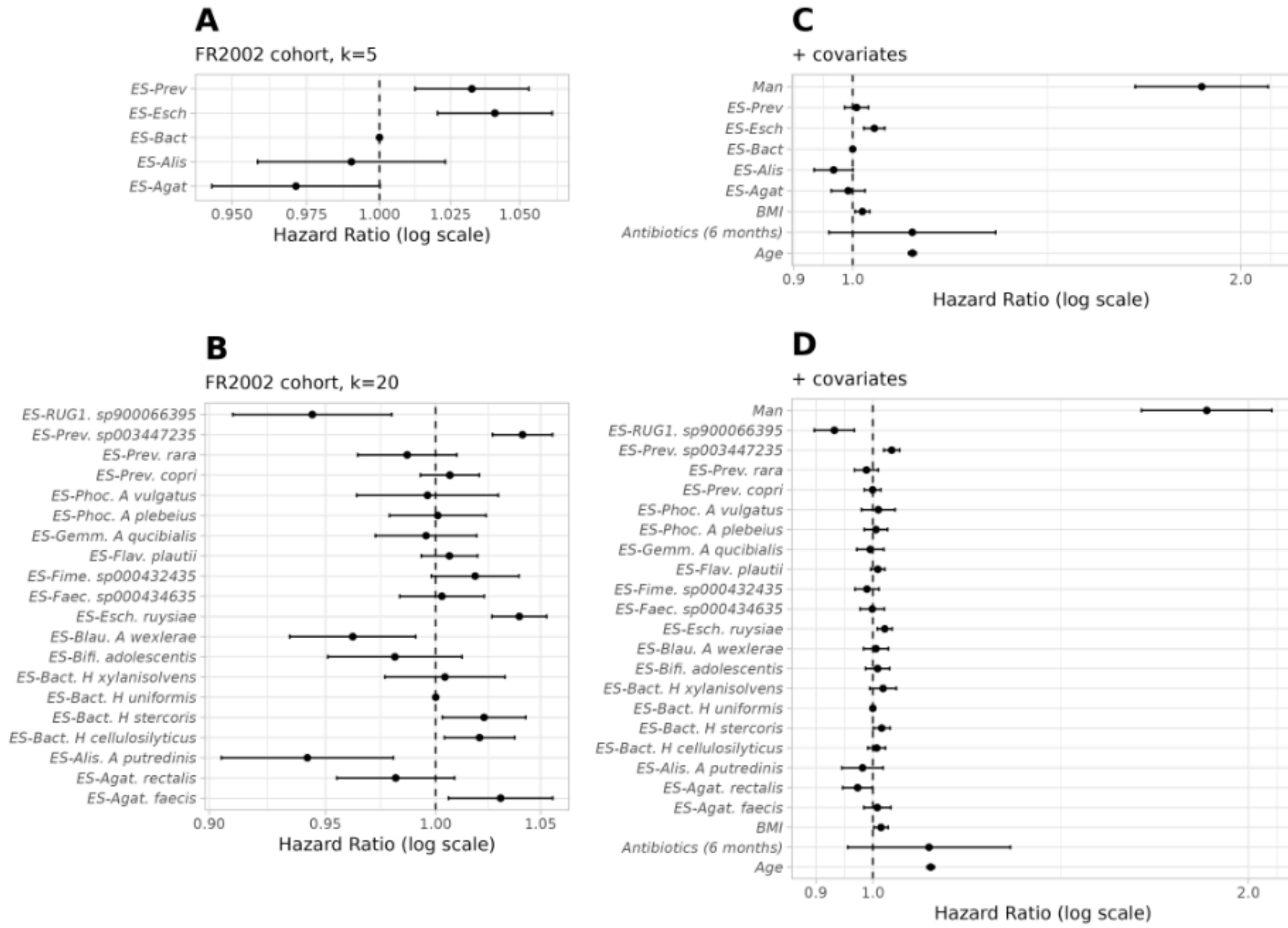

Figure S3. Cox proportional hazards regression, multivariate (mortality) using only FINRISK2002 cohort. The hazard ratios (HR) for the signatures are reported per 10^6-unit increase in the predictor. **A:** The multivariate effect sizes of the signatures, k=5. *ES-Prev* and *ES-Esch* are associated with higher risk for mortality. **B:** The multivariate effect sizes of the signatures, k=20. *ES-Prev. sp003447235*, *ES-Esch. ruysiae*, *ES-Bact. cellulosilyticus*, *ES-Agat. faecis* are associated with higher risk for mortality (q-value<0.05, Table S6), and *ES-RUG115 sp900066395*, *ES-Blau. A wexlerae*, and *ES-Alis. A putredinis* with lower (q-value<0.05, Table S6). **C:** The multivariate effect sizes of the signatures, k=5, adjusted with covariates (sex, age, BMI, antibiotic use). *ES-Esch* is associated with higher risk for mortality. **D:** The multivariate effect sizes of the signatures, k=20, adjusted with covariates. *ES-Prev. sp003447235* and *ES-Esch. ruysiae* are associated with higher risk for mortality, and *ES-RUG115 sp900066395* with lower.

Table S1. Multivariate Cox proportional hazards regression, continuous predictors (* q<0.05; ** q<0.01; ***q<0.001). The column *k* represents the level of granularity (*k*={1,2,...,20} for mortality endpoint; *k*={5,20} for diabetes, liver disease and sepsis, each). At *k*=5 the signatures are named by the highest indicator species; *ES-Gemmiger A 73129* represents *ES-Alis*, *ES-Bacteroides H cellulosilyticus ES-Esch*, *ES-Agathobacter rectalis ES-Agat*, *ES-Prevotella copri ES-Prev*, and *ES-Bacteroides H uniformis ES-Bact*.

endpoint	k	name	HR	95%CI lower	95%CI upper	q	significant
Death	1	ES-Bacteroides H uniformis	0.99	0.94	1.04	8.4×10^{-1}	
Death	2	ES-Prevotella copri	1.00	0.97	1.04	9.5×10^{-1}	
Death	2	ES-Bacteroides H uniformis	0.98	0.94	1.03	6.5×10^{-1}	
Death	3	ES-Agathobacter rectalis	0.96	0.93	1.00	1.2×10^{-1}	
Death	3	ES-Prevotella copri	1.07	1.05	1.10	1.1×10^{-6}	***
Death	3	ES-Bacteroides H uniformis	0.99	0.95	1.03	8.4×10^{-1}	
Death	4	ES-Bacteroides H uniformis	0.99	0.95	1.03	7.5×10^{-1}	
Death	4	ES-Gemmiger A 73129 qucibialis	1.02	0.99	1.06	3.3×10^{-1}	
Death	4	ES-Prevotella copri	1.03	1.01	1.05	3.8×10^{-2}	*
Death	4	ES-Agathobacter rectalis	0.97	0.94	1.00	2.1×10^{-1}	
Death	5	ES-Gemmiger A 73129 qucibialis	0.99	0.96	1.03	8.8×10^{-1}	
Death	5	ES-Bacteroides H cellulosilyticus	1.04	1.02	1.06	7.2×10^{-4}	***
Death	5	ES-Agathobacter rectalis	0.97	0.94	1.00	1.5×10^{-1}	
Death	5	ES-Prevotella copri	1.03	1.01	1.05	1.1×10^{-2}	*
Death	5	ES-Bacteroides H uniformis	0.98	0.94	1.01	4.2×10^{-1}	
Death	6	ES-Gemmiger A 73129 qucibialis	1.00	0.97	1.03	9.7×10^{-1}	
Death	6	ES-Prevotella copri	1.03	1.01	1.05	1.6×10^{-2}	*
Death	6	ES-Bacteroides H uniformis	0.98	0.95	1.01	4.4×10^{-1}	
Death	6	ES-Agathobacter rectalis	0.97	0.94	0.99	7.2×10^{-2}	
Death	6	ES-Bacteroides H stercoris	1.01	0.98	1.04	6.6×10^{-1}	
Death	6	ES-Bacteroides H fragilis	1.03	1.02	1.05	1.4×10^{-3}	**
Death	7	ES-Agathobacter rectalis	0.96	0.93	0.99	5.3×10^{-2}	
Death	7	ES-Gemmiger A 73129 qucibialis	1.00	0.97	1.03	9.5×10^{-1}	
Death	7	ES-Prevotella copri	1.03	1.01	1.05	1.7×10^{-2}	*
Death	7	ES-Bacteroides H cellulosilyticus	1.00	0.97	1.03	9.6×10^{-1}	
Death	7	ES-Bifidobacterium adolescentis	1.03	1.01	1.04	7.0×10^{-3}	**
Death	7	ES-Bacteroides H stercoris	1.01	0.98	1.04	6.7×10^{-1}	
Death	7	ES-Bacteroides H uniformis	0.98	0.95	1.02	5.2×10^{-1}	
Death	8	ES-Bacteroides H cellulosilyticus	0.97	0.94	1.01	2.3×10^{-1}	
Death	8	ES-Prevotella copri	1.02	1.00	1.04	1.1×10^{-1}	
Death	8	ES-Bacteroides H fragilis	1.04	1.02	1.05	6.6×10^{-5}	***
Death	8	ES-Bifidobacterium adolescentis	0.96	0.93	1.00	8.8×10^{-2}	
Death	8	ES-Bacteroides H uniformis	0.99	0.96	1.02	7.3×10^{-1}	
Death	8	ES-Gemmiger A 73129 qucibialis	1.03	1.00	1.06	8.1×10^{-2}	
Death	8	ES-Agathobacter rectalis	0.99	0.96	1.01	5.5×10^{-1}	
Death	8	ES-Bacteroides H stercoris	1.01	0.98	1.04	6.9×10^{-1}	
Death	9	ES-Phocaeicola A 858004 plebeius	1.00	0.97	1.02	9.5×10^{-1}	
Death	9	ES-Fimenecus sp000432435	1.05	1.03	1.07	7.0×10^{-5}	***
Death	9	ES-Prevotella copri	1.01	0.99	1.03	4.7×10^{-1}	
Death	9	ES-Alistipes A 871400 putredinis	0.96	0.93	0.99	4.9×10^{-2}	*
Death	9	ES-Agathobacter rectalis	0.98	0.95	1.00	2.3×10^{-1}	
Death	9	ES-Bacteroides H cellulosilyticus	1.04	1.02	1.05	1.8×10^{-6}	***
Death	9	ES-Bacteroides H uniformis	1.01	0.98	1.05	7.5×10^{-1}	
Death	9	ES-Bifidobacterium adolescentis	0.98	0.96	1.01	3.7×10^{-1}	
Death	9	ES-Phocaeicola A 858004 vulgatus	0.98	0.94	1.01	4.4×10^{-1}	
Death	10	ES-Bacteroides H cellulosilyticus	0.97	0.94	1.00	2.1×10^{-1}	
Death	10	ES-Prevotella copri	1.02	1.00	1.04	6.1×10^{-2}	
Death	10	ES-Agathobacter faecis	1.00	0.97	1.03	9.9×10^{-1}	
Death	10	ES-Bacteroides H fragilis	1.03	1.02	1.04	1.5×10^{-4}	***
Death	10	ES-Bifidobacterium adolescentis	0.97	0.95	1.00	1.6×10^{-1}	
Death	10	ES-Phocaeicola A 858004 vulgatus	1.01	0.98	1.04	7.5×10^{-1}	
Death	10	ES-Phocaeicola A 858004 plebeius	1.00	0.97	1.02	9.1×10^{-1}	
Death	10	ES-Fimenecus sp000432435	1.03	1.01	1.06	5.3×10^{-2}	
Death	10	ES-Agathobacter rectalis	0.97	0.94	1.00	1.9×10^{-1}	
Death	10	ES-Bacteroides H uniformis	0.99	0.96	1.03	8.4×10^{-1}	
Death	11	ES-Bacteroides H cellulosilyticus	1.01	0.98	1.03	8.6×10^{-1}	
Death	11	ES-Agathobacter faecis	0.99	0.96	1.03	9.0×10^{-1}	
Death	11	ES-Agathobacter rectalis	0.97	0.94	1.00	1.3×10^{-1}	
Death	11	ES-Fimenecus sp000432435	1.05	1.04	1.07	3.8×10^{-8}	***
Death	11	ES-Escherichia ruysiae	1.02	1.01	1.04	2.2×10^{-5}	***
Death	11	ES-Prevotella copri	1.01	0.99	1.02	5.9×10^{-1}	
Death	11	ES-Bifidobacterium adolescentis	0.98	0.95	1.00	1.5×10^{-1}	
Death	11	ES-Bacteroides H uniformis	1.00	0.96	1.03	9.1×10^{-1}	
Death	11	ES-Alistipes A 871400 putredinis	0.97	0.94	1.00	1.0×10^{-1}	

Death	11	ES-Phocaeicola A 858004 plebeius	1.00	0.97	1.02	9.1×10^{-1}	
Death	11	ES-Phocaeicola A 858004 vulgatus	1.00	0.97	1.04	9.5×10^{-1}	
Death	12	ES-Alistipes A 871400 putredinis	0.97	0.94	1.00	9.8×10^{-2}	
Death	12	ES-Agathobacter faecis	1.00	0.97	1.03	9.2×10^{-1}	
Death	12	ES-Phocaeicola A 858004 plebeius	1.00	0.97	1.02	8.8×10^{-1}	
Death	12	ES-Bacteroides H cellulosilyticus	1.01	0.99	1.03	5.7×10^{-1}	
Death	12	ES-Bacteroides H stercoris	1.03	1.00	1.05	1.2×10^{-1}	
Death	12	ES-Bifidobacterium adolescentis	0.98	0.95	1.00	1.6×10^{-1}	
Death	12	ES-Phocaeicola A 858004 vulgatus	0.99	0.95	1.02	5.8×10^{-1}	
Death	12	ES-Bacteroides H uniformis	1.00	0.96	1.03	9.1×10^{-1}	
Death	12	ES-Prevotella copri	1.01	0.99	1.02	5.7×10^{-1}	
Death	12	ES-Bacteroides H fragilis	1.02	1.01	1.04	4.5×10^{-5}	***
Death	12	ES-Fimenecus sp000432435	1.05	1.03	1.07	5.2×10^{-8}	***
Death	12	ES-Agathobacter rectalis	0.97	0.94	1.00	1.6×10^{-1}	
Death	13	ES-Bacteroides H cellulosilyticus	1.01	1.00	1.03	2.9×10^{-1}	
Death	13	ES-Agathobacter rectalis	0.97	0.94	1.00	1.4×10^{-1}	
Death	13	ES-Phocaeicola A 858004 plebeius	1.00	0.97	1.02	8.7×10^{-1}	
Death	13	ES-Blautia A 141781 wexlerae	1.00	0.98	1.02	9.4×10^{-1}	
Death	13	ES-Bacteroides H fragilis	1.04	1.03	1.05	2.0×10^{-7}	***
Death	13	ES-Bacteroides H stercoris	1.02	1.00	1.05	1.6×10^{-1}	
Death	13	ES-Fimenecus sp000432435	1.05	1.03	1.07	4.1×10^{-6}	***
Death	13	ES-Bacteroides H uniformis	0.99	0.96	1.03	9.1×10^{-1}	
Death	13	ES-Prevotella copri	1.01	1.00	1.03	3.5×10^{-1}	
Death	13	ES-Agathobacter faecis	1.00	0.97	1.03	9.9×10^{-1}	
Death	13	ES-Gemmiger A 73129 qucibialis	0.98	0.96	1.01	4.2×10^{-1}	
Death	13	ES-Alistipes A 871400 putredinis	0.96	0.94	0.99	6.1×10^{-2}	
Death	13	ES-Phocaeicola A 858004 vulgatus	0.99	0.96	1.02	7.8×10^{-1}	
Death	14	ES-Blautia A 141781 wexlerae	0.97	0.95	1.00	7.5×10^{-2}	
Death	14	ES-Fimenecus sp000432435	1.03	1.02	1.05	1.6×10^{-3}	**
Death	14	ES-Alistipes A 871400 putredinis	0.92	0.89	0.96	7.2×10^{-4}	***
Death	14	ES-Prevotella copri	1.01	1.00	1.03	1.9×10^{-1}	
Death	14	ES-Agathobacter faecis	1.02	0.99	1.05	3.5×10^{-1}	
Death	14	ES-Gemmiger A 73129 qucibialis	0.99	0.97	1.02	8.6×10^{-1}	
Death	14	ES-Phocaeicola A 858004 vulgatus	1.02	0.99	1.05	3.2×10^{-1}	
Death	14	ES-Bacteroides H cellulosilyticus	1.02	1.00	1.04	9.1×10^{-2}	
Death	14	ES-Bifidobacterium adolescentis	0.99	0.96	1.02	6.2×10^{-1}	
Death	14	ES-Bacteroides H xylanisolvens	1.00	0.97	1.03	9.6×10^{-1}	
Death	14	ES-Phocaeicola A 858004 plebeius	1.00	0.98	1.02	9.5×10^{-1}	
Death	14	ES-Escherichia ruysiae	1.05	1.04	1.06	1.5×10^{-11}	***
Death	14	ES-Bacteroides H uniformis	1.00	0.97	1.04	9.6×10^{-1}	
Death	14	ES-Agathobacter rectalis	0.97	0.95	1.00	1.9×10^{-1}	
Death	15	ES-Escherichia ruysiae	1.05	1.03	1.06	9.1×10^{-12}	***
Death	15	ES-Agathobacter faecis	1.02	0.99	1.05	4.7×10^{-1}	
Death	15	ES-Bacteroides H cellulosilyticus	1.02	1.00	1.04	1.0×10^{-1}	
Death	15	ES-Alistipes A 871400 putredinis	0.95	0.92	0.98	7.3×10^{-3}	**
Death	15	ES-Blautia A 141781 wexlerae	0.98	0.96	1.00	1.5×10^{-1}	
Death	15	ES-Agathobacter rectalis	0.97	0.94	1.00	1.5×10^{-1}	
Death	15	ES-Gemmiger A 73129 qucibialis	1.00	0.98	1.02	9.5×10^{-1}	
Death	15	ES-Bacteroides H stercoris	1.03	1.00	1.05	8.3×10^{-2}	
Death	15	ES-Prevotella sp003447235	1.03	1.02	1.05	5.3×10^{-4}	***
Death	15	ES-Prevotella copri	1.01	1.00	1.03	2.0×10^{-1}	
Death	15	ES-Bifidobacterium adolescentis	0.99	0.97	1.02	8.4×10^{-1}	
Death	15	ES-Bacteroides H xylanisolvens	0.99	0.96	1.03	8.4×10^{-1}	
Death	15	ES-Phocaeicola A 858004 vulgatus	1.00	0.96	1.03	9.7×10^{-1}	
Death	15	ES-Phocaeicola A 858004 plebeius	1.00	0.97	1.02	8.6×10^{-1}	
Death	15	ES-Bacteroides H uniformis	1.00	0.97	1.03	9.9×10^{-1}	
Death	16	ES-Gemmiger A 73129 qucibialis	1.00	0.97	1.03	9.6×10^{-1}	
Death	16	ES-Fimenecus sp000432435	1.03	1.01	1.05	4.5×10^{-2}	*
Death	16	ES-Agathobacter rectalis	0.99	0.96	1.02	6.0×10^{-1}	
Death	16	ES-Bacteroides H cellulosilyticus	1.02	1.00	1.04	1.9×10^{-1}	
Death	16	ES-Alistipes A 871400 putredinis	0.93	0.89	0.96	1.6×10^{-3}	**
Death	16	ES-Blautia A 141781 wexlerae	0.97	0.94	0.99	4.1×10^{-2}	*
Death	16	ES-Prevotella copri	1.01	1.00	1.03	1.6×10^{-1}	
Death	16	ES-Bacteroides H uniformis	1.00	0.97	1.03	9.9×10^{-1}	
Death	16	ES-Phocaeicola A 858004 plebeius	1.00	0.97	1.02	9.5×10^{-1}	
Death	16	ES-Bifidobacterium adolescentis	0.99	0.97	1.02	8.1×10^{-1}	
Death	16	ES-Bacteroides H fragilis	1.01	1.00	1.03	2.3×10^{-1}	
Death	16	ES-Phocaeicola A 858004 vulgatus	1.00	0.96	1.03	9.1×10^{-1}	
Death	16	ES-Agathobacter faecis	1.01	0.98	1.04	6.9×10^{-1}	
Death	16	ES-Escherichia ruysiae	1.04	1.03	1.06	1.4×10^{-7}	***
Death	16	ES-Faecousia sp000434635	1.02	1.00	1.04	2.5×10^{-1}	
Death	16	ES-Bacteroides H stercoris	1.03	1.01	1.05	4.0×10^{-2}	*
Death	17	ES-Bifidobacterium adolescentis	0.97	0.94	1.01	2.9×10^{-1}	
Death	17	ES-Escherichia ruysiae	1.04	1.03	1.06	5.7×10^{-8}	***
Death	17	ES-Bacteroides H uniformis	1.00	0.97	1.03	9.7×10^{-1}	
Death	17	ES-Dialister invisus	1.00	0.99	1.02	7.5×10^{-1}	

Death	17	ES-Prevotella copri	1.01	0.99	1.02	5.3×10^{-1}	
Death	17	ES-Faecousia sp000434635	1.01	0.98	1.03	8.4×10^{-1}	
Death	17	ES-Phocaeicola A 858004 plebeius	1.00	0.98	1.01	8.4×10^{-1}	
Death	17	ES-Phocaeicola A 858004 vulgatus	1.00	0.97	1.03	9.7×10^{-1}	
Death	17	ES-Bacteroides H stercoris	1.02	1.00	1.05	1.9×10^{-1}	
Death	17	ES-Prevotella sp003447235	1.05	1.03	1.06	5.7×10^{-8}	***
Death	17	ES-Bacteroides H cellulosilyticus	1.02	1.00	1.04	7.9×10^{-2}	
Death	17	ES-RUG115 sp900066395	0.95	0.91	0.98	1.2×10^{-2}	*
Death	17	ES-Agathobacter rectalis	0.97	0.94	1.00	1.3×10^{-1}	
Death	17	ES-Gemmiger A 73129 qucibialis	1.01	0.99	1.03	7.3×10^{-1}	
Death	17	ES-Alistipes A 871400 putredinis	0.94	0.90	0.98	1.5×10^{-2}	*
Death	17	ES-Blautia A 141781 wexlerae	0.97	0.94	1.00	1.4×10^{-1}	
Death	17	ES-Agathobacter faecis	1.03	1.00	1.06	1.1×10^{-1}	
Death	18	ES-Gemmiger A 73129 qucibialis	0.99	0.97	1.02	8.4×10^{-1}	
Death	18	ES-Dialister invisus	1.01	0.99	1.02	5.9×10^{-1}	
Death	18	ES-Bifidobacterium adolescentis	0.99	0.96	1.01	5.6×10^{-1}	
Death	18	ES-Prevotella copri	1.01	0.99	1.02	5.0×10^{-1}	
Death	18	ES-Agathobacter rectalis	0.98	0.95	1.01	2.5×10^{-1}	
Death	18	ES-Escherichia ruysiae	1.04	1.03	1.05	1.0×10^{-8}	***
Death	18	ES-Bacteroides H cellulosilyticus	1.02	1.00	1.03	1.0×10^{-1}	
Death	18	ES-Fimenecus sp000432435	1.05	1.03	1.06	2.4×10^{-6}	***
Death	18	ES-Bacteroides H xylanisolvens	1.00	0.97	1.03	9.1×10^{-1}	
Death	18	ES-Bacteroides H stercoris	1.03	1.00	1.05	8.3×10^{-2}	
Death	18	ES-Alistipes A 871400 putredinis	0.94	0.91	0.98	1.5×10^{-2}	*
Death	18	ES-Faecousia sp000434635	1.00	0.97	1.02	9.2×10^{-1}	
Death	18	ES-Phocaeicola A 858004 plebeius	1.00	0.98	1.02	9.1×10^{-1}	
Death	18	ES-Blautia A 141781 wexlerae	0.98	0.95	1.01	2.5×10^{-1}	
Death	18	ES-Phocaeicola A 858004 vulgatus	1.00	0.96	1.03	9.5×10^{-1}	
Death	18	ES-Bacteroides H uniformis	1.00	0.97	1.03	9.5×10^{-1}	
Death	18	ES-Agathobacter faecis	1.02	0.99	1.05	4.7×10^{-1}	
Death	18	ES-RUG115 sp900066395	0.97	0.94	1.00	1.5×10^{-1}	
Death	19	ES-Bacteroides H xylanisolvens	0.99	0.96	1.02	8.4×10^{-1}	
Death	19	ES-Bacteroides H uniformis	1.00	0.97	1.03	9.7×10^{-1}	
Death	19	ES-Gemmiger A 73129 qucibialis	1.01	0.99	1.03	6.9×10^{-1}	
Death	19	ES-Butyrivibrio A 180067 crossotus	1.03	1.02	1.05	8.8×10^{-7}	***
Death	19	ES-Phocaeicola A 858004 plebeius	1.00	0.98	1.02	8.4×10^{-1}	
Death	19	ES-Agathobacter rectalis	0.98	0.95	1.01	4.1×10^{-1}	
Death	19	ES-Alistipes A 871400 putredinis	0.94	0.91	0.98	1.7×10^{-2}	*
Death	19	ES-Escherichia ruysiae	1.04	1.03	1.05	3.0×10^{-8}	***
Death	19	ES-Agathobacter faecis	1.03	1.01	1.06	4.0×10^{-2}	*
Death	19	ES-Blautia A 141781 wexlerae	0.96	0.93	0.99	7.2×10^{-2}	
Death	19	ES-RUG115 sp900066395	0.94	0.91	0.97	6.3×10^{-3}	**
Death	19	ES-Bacteroides H stercoris	1.03	1.01	1.05	6.1×10^{-2}	
Death	19	ES-Prevotella copri	1.01	1.00	1.02	3.6×10^{-1}	
Death	19	ES-Bacteroides H cellulosilyticus	1.02	1.00	1.04	6.4×10^{-2}	
Death	19	ES-Faecousia sp000434635	1.00	0.98	1.02	9.1×10^{-1}	
Death	19	ES-Parabacteroides B 862066 distasonis	1.01	0.97	1.05	9.1×10^{-1}	
Death	19	ES-Phocaeicola A 858004 vulgatus	1.01	0.97	1.04	9.1×10^{-1}	
Death	19	ES-Dialister invisus	1.00	0.99	1.02	7.3×10^{-1}	
Death	19	ES-Bifidobacterium adolescentis	0.98	0.95	1.01	3.1×10^{-1}	
Death	20	ES-Blautia A 141781 wexlerae	0.96	0.93	0.99	5.3×10^{-2}	
Death	20	ES-Alistipes A 871400 putredinis	0.94	0.90	0.98	1.9×10^{-2}	*
Death	20	ES-Agathobacter rectalis	0.98	0.95	1.01	2.5×10^{-1}	
Death	20	ES-Bifidobacterium adolescentis	0.98	0.95	1.01	3.7×10^{-1}	
Death	20	ES-Phocaeicola A 858004 vulgatus	1.00	0.97	1.03	9.5×10^{-1}	
Death	20	ES-Fimenecus sp000432435	1.02	1.00	1.04	1.4×10^{-1}	
Death	20	ES-Bacteroides H cellulosilyticus	1.02	1.00	1.04	6.6×10^{-2}	
Death	20	ES-RUG115 sp900066395	0.94	0.91	0.98	1.3×10^{-2}	*
Death	20	ES-Faecousia sp000434635	1.00	0.98	1.02	8.8×10^{-1}	
Death	20	ES-Phocaeicola A 858004 plebeius	1.00	0.98	1.02	9.5×10^{-1}	
Death	20	ES-Prevotella rara	0.99	0.97	1.01	5.0×10^{-1}	
Death	20	ES-Escherichia ruysiae	1.04	1.03	1.05	3.3×10^{-8}	***
Death	20	ES-Agathobacter faecis	1.03	1.01	1.06	5.3×10^{-2}	
Death	20	ES-Bacteroides H stercoris	1.02	1.00	1.04	9.9×10^{-2}	
Death	20	ES-Bacteroides H xylanisolvens	1.00	0.97	1.03	9.5×10^{-1}	
Death	20	ES-Flavonifractor plautii	1.01	0.99	1.02	5.1×10^{-1}	
Death	20	ES-Prevotella copri	1.01	0.99	1.02	6.0×10^{-1}	
Death	20	ES-Gemmiger A 73129 qucibialis	1.00	0.97	1.02	9.1×10^{-1}	
Death	20	ES-Prevotella sp003447235	1.04	1.03	1.06	2.0×10^{-7}	***
Death	20	ES-Bacteroides H uniformis	1.00	0.97	1.03	9.7×10^{-1}	
Diabetes	5	ES-Gemmiger A 73129 qucibialis	0.92	0.88	0.96	6.4×10^{-4}	***
Diabetes	5	ES-Bacteroides H cellulosilyticus	1.01	0.98	1.04	8.1×10^{-1}	
Diabetes	5	ES-Agathobacter rectalis	1.05	1.02	1.07	2.3×10^{-4}	***
Diabetes	5	ES-Prevotella copri	1.04	1.02	1.06	2.3×10^{-4}	***
Diabetes	5	ES-Bacteroides H uniformis	1.02	0.99	1.05	4.9×10^{-1}	
Diabetes	20	ES-Blautia A 141781 wexlerae	1.02	1.00	1.03	8.6×10^{-2}	

Diabetes	20	ES-Alistipes A 871400 putredinis	0.93	0.89	0.97	4.2×10^{-3}	**
Diabetes	20	ES-Agathobacter rectalis	1.01	0.99	1.03	6.4×10^{-1}	
Diabetes	20	ES-Bifidobacterium adolescentis	0.97	0.94	1.00	2.1×10^{-1}	
Diabetes	20	ES-Phocaeicola A 858004 vulgatus	1.03	1.01	1.05	4.8×10^{-2}	*
Diabetes	20	ES-Fimenecus sp000432435	1.02	0.99	1.04	2.9×10^{-1}	
Diabetes	20	ES-Bacteroides H cellulosilyticus	1.00	0.97	1.03	9.7×10^{-1}	
Diabetes	20	ES-RUG115 sp900066395	0.97	0.94	1.00	2.0×10^{-1}	
Diabetes	20	ES-Faecousia sp000434635	0.97	0.93	1.00	1.2×10^{-1}	
Diabetes	20	ES-Phocaeicola A 858004 plebeius	1.00	0.98	1.02	9.7×10^{-1}	
Diabetes	20	ES-Prevotella rara	1.00	0.98	1.02	9.2×10^{-1}	
Diabetes	20	ES-Escherichia ruysiae	1.01	0.98	1.03	7.4×10^{-1}	
Diabetes	20	ES-Agathobacter faecis	1.02	0.99	1.04	2.9×10^{-1}	
Diabetes	20	ES-Bacteroides H stercoris	1.02	1.00	1.04	1.3×10^{-1}	
Diabetes	20	ES-Bacteroides H xylanisolvens	0.99	0.97	1.02	8.9×10^{-1}	
Diabetes	20	ES-Flavonifractor plautii	1.01	1.00	1.02	2.3×10^{-1}	
Diabetes	20	ES-Prevotella copri	1.02	1.01	1.04	3.6×10^{-3}	**
Diabetes	20	ES-Gemmiger A 73129 qucibialis	1.01	0.99	1.03	7.2×10^{-1}	
Diabetes	20	ES-Prevotella sp003447235	1.00	0.98	1.02	9.5×10^{-1}	
Diabetes	20	ES-Bacteroides H uniformis	1.01	0.98	1.03	8.4×10^{-1}	
Liver dis.	5	ES-Gemmiger A 73129 qucibialis	0.73	0.61	0.87	4.2×10^{-3}	**
Liver dis.	5	ES-Bacteroides H cellulosilyticus	1.07	1.02	1.11	1.5×10^{-2}	*
Liver dis.	5	ES-Agathobacter rectalis	1.00	0.93	1.06	9.5×10^{-1}	
Liver dis.	5	ES-Prevotella copri	0.96	0.86	1.06	6.3×10^{-1}	
Liver dis.	5	ES-Bacteroides H uniformis	1.01	0.93	1.10	9.3×10^{-1}	
Liver dis.	20	ES-Blautia A 141781 wexlerae	1.02	0.99	1.05	3.3×10^{-1}	
Liver dis.	20	ES-Alistipes A 871400 putredinis	0.89	0.78	1.01	1.9×10^{-1}	
Liver dis.	20	ES-Agathobacter rectalis	1.02	0.95	1.08	8.4×10^{-1}	
Liver dis.	20	ES-Bifidobacterium adolescentis	1.01	0.95	1.07	9.1×10^{-1}	
Liver dis.	20	ES-Phocaeicola A 858004 vulgatus	1.06	0.99	1.13	2.3×10^{-1}	
Liver dis.	20	ES-Fimenecus sp000432435	1.02	0.95	1.11	7.6×10^{-1}	
Liver dis.	20	ES-Bacteroides H cellulosilyticus	0.97	0.88	1.06	7.3×10^{-1}	
Liver dis.	20	ES-RUG115 sp900066395	0.79	0.66	0.94	4.0×10^{-2}	*
Liver dis.	20	ES-Faecousia sp000434635	0.97	0.87	1.07	7.5×10^{-1}	
Liver dis.	20	ES-Phocaeicola A 858004 plebeius	1.02	0.97	1.07	7.3×10^{-1}	
Liver dis.	20	ES-Prevotella rara	0.89	0.75	1.05	3.5×10^{-1}	
Liver dis.	20	ES-Escherichia ruysiae	1.04	1.01	1.08	2.6×10^{-2}	*
Liver dis.	20	ES-Agathobacter faecis	0.94	0.86	1.03	3.5×10^{-1}	
Liver dis.	20	ES-Bacteroides H stercoris	1.04	1.01	1.08	9.5×10^{-2}	
Liver dis.	20	ES-Bacteroides H xylanisolvens	0.99	0.92	1.07	9.5×10^{-1}	
Liver dis.	20	ES-Flavonifractor plautii	1.01	0.99	1.03	4.9×10^{-1}	
Liver dis.	20	ES-Prevotella copri	0.99	0.93	1.04	8.4×10^{-1}	
Liver dis.	20	ES-Gemmiger A 73129 qucibialis	0.87	0.75	1.01	1.7×10^{-1}	
Liver dis.	20	ES-Prevotella sp003447235	0.98	0.92	1.06	8.6×10^{-1}	
Liver dis.	20	ES-Bacteroides H uniformis	1.01	0.94	1.08	9.5×10^{-1}	
Sepsis	5	ES-Gemmiger A 73129 qucibialis	1.03	0.98	1.10	4.5×10^{-1}	
Sepsis	5	ES-Bacteroides H cellulosilyticus	1.06	1.02	1.11	1.2×10^{-2}	*
Sepsis	5	ES-Agathobacter rectalis	0.96	0.89	1.02	3.8×10^{-1}	
Sepsis	5	ES-Prevotella copri	1.04	1.00	1.08	1.6×10^{-1}	
Sepsis	5	ES-Bacteroides H uniformis	0.98	0.90	1.06	8.1×10^{-1}	
Sepsis	20	ES-Blautia A 141781 wexlerae	0.93	0.87	1.00	1.4×10^{-1}	
Sepsis	20	ES-Alistipes A 871400 putredinis	0.97	0.90	1.05	7.3×10^{-1}	
Sepsis	20	ES-Agathobacter rectalis	0.95	0.89	1.02	3.1×10^{-1}	
Sepsis	20	ES-Bifidobacterium adolescentis	1.01	0.96	1.06	9.4×10^{-1}	
Sepsis	20	ES-Phocaeicola A 858004 vulgatus	0.99	0.93	1.05	9.1×10^{-1}	
Sepsis	20	ES-Fimenecus sp000432435	1.03	0.98	1.07	4.5×10^{-1}	
Sepsis	20	ES-Bacteroides H cellulosilyticus	0.93	0.85	1.02	2.3×10^{-1}	
Sepsis	20	ES-RUG115 sp900066395	1.02	0.97	1.07	7.4×10^{-1}	
Sepsis	20	ES-Faecousia sp000434635	1.02	0.98	1.06	5.7×10^{-1}	
Sepsis	20	ES-Phocaeicola A 858004 plebeius	0.98	0.92	1.04	7.5×10^{-1}	
Sepsis	20	ES-Prevotella rara	1.01	0.97	1.05	8.4×10^{-1}	
Sepsis	20	ES-Escherichia ruysiae	1.03	1.00	1.07	1.4×10^{-1}	
Sepsis	20	ES-Agathobacter faecis	1.02	0.97	1.08	6.0×10^{-1}	
Sepsis	20	ES-Bacteroides H stercoris	0.92	0.82	1.02	2.3×10^{-1}	
Sepsis	20	ES-Bacteroides H xylanisolvens	1.01	0.96	1.06	9.4×10^{-1}	
Sepsis	20	ES-Flavonifractor plautii	1.04	1.02	1.06	6.5×10^{-3}	**
Sepsis	20	ES-Prevotella copri	1.00	0.97	1.03	9.7×10^{-1}	
Sepsis	20	ES-Gemmiger A 73129 qucibialis	0.99	0.93	1.04	8.4×10^{-1}	
Sepsis	20	ES-Prevotella sp003447235	1.03	1.00	1.06	1.9×10^{-1}	
Sepsis	20	ES-Bacteroides H uniformis	1.04	0.99	1.09	2.3×10^{-1}	

Table S2. Kendall's tau correlation analysis for the signatures and selected variables. Unadjusted p-values are shown; the stars denote the level (* p<0.05; ** p<0.01; *** p<0.001)

ES	variable	tau	p	significant
ES-Alis	Age	0.07	2.1×10^{-16}	***
ES-Alis	Man	-0.07	6.4×10^{-12}	***
ES-Alis	BMI	-0.08	1.1×10^{-23}	***
ES-Alis	Vegetarian	-0.01	1.6×10^{-1}	
ES-Alis	Prevalent diabetes	0.00	6.2×10^{-1}	
ES-Alis	Prior liver disease	0.00	7.7×10^{-1}	
ES-Alis	Prior sepsis	-0.01	2.9×10^{-1}	
ES-Alis	Pregnant	0.02	1.2×10^{-1}	
ES-Alis	Antibiotics (6 months)	-0.08	6.9×10^{-16}	***
ES-Esch	Age	-0.02	1.7×10^{-2}	*
ES-Esch	Man	-0.10	5.6×10^{-27}	***
ES-Esch	BMI	-0.05	3.4×10^{-9}	***
ES-Esch	Vegetarian	0.01	2.2×10^{-1}	
ES-Esch	Prevalent diabetes	0.05	2.7×10^{-7}	***
ES-Esch	Prior liver disease	0.02	4.3×10^{-2}	*
ES-Esch	Prior sepsis	0.02	3.7×10^{-2}	*
ES-Esch	Pregnant	0.00	8.8×10^{-1}	
ES-Esch	Antibiotics (6 months)	0.12	5.5×10^{-37}	***
ES-Agat	Age	-0.06	1.3×10^{-12}	***
ES-Agat	Man	0.06	1.2×10^{-8}	***
ES-Agat	BMI	0.07	8.2×10^{-19}	***
ES-Agat	Vegetarian	-0.01	3.2×10^{-1}	
ES-Agat	Prevalent diabetes	-0.03	3.4×10^{-3}	**
ES-Agat	Prior liver disease	0.00	6.8×10^{-1}	
ES-Agat	Prior sepsis	0.00	7.1×10^{-1}	
ES-Agat	Pregnant	0.00	8.7×10^{-1}	
ES-Agat	Antibiotics (6 months)	-0.01	4.2×10^{-1}	
ES-Prev	Age	0.07	2.0×10^{-19}	***
ES-Prev	Man	0.11	7.0×10^{-31}	***
ES-Prev	BMI	0.06	5.7×10^{-13}	***
ES-Prev	Vegetarian	0.00	7.9×10^{-1}	
ES-Prev	Prevalent diabetes	0.01	2.0×10^{-1}	
ES-Prev	Prior liver disease	0.00	9.3×10^{-1}	
ES-Prev	Prior sepsis	-0.02	5.6×10^{-2}	
ES-Prev	Pregnant	0.00	7.2×10^{-1}	
ES-Prev	Antibiotics (6 months)	-0.08	2.3×10^{-15}	***
ES-Bact	Age	-0.08	6.3×10^{-23}	***
ES-Bact	Man	-0.04	1.6×10^{-4}	***
ES-Bact	BMI	0.01	8.0×10^{-2}	
ES-Bact	Vegetarian	-0.01	1.8×10^{-1}	
ES-Bact	Prevalent diabetes	0.01	3.4×10^{-1}	
ES-Bact	Prior liver disease	0.01	2.7×10^{-1}	
ES-Bact	Prior sepsis	-0.02	5.8×10^{-2}	
ES-Bact	Pregnant	-0.01	4.3×10^{-1}	
ES-Bact	Antibiotics (6 months)	0.05	5.7×10^{-7}	***

Table S3. Categorical Cox proportional hazards regression (k=5), where *ES-Bact* is set as the reference level (* q<0.05; ** q<0.01; ***q<0.001).

principal ES	HR	95%CI lower	95%CI upper	q	significant
ES-Agat	1.12	0.94	1.33	3.0×10^{-1}	
ES-Alis	1.09	0.93	1.28	3.0×10^{-1}	
ES-Esch	1.47	1.21	1.79	2.5×10^{-4}	***
ES-Prev	1.48	1.21	1.79	2.5×10^{-4}	***

Table S4. Categorical Cox proportional hazards regression (k=20), where *ES-Bact. H uniformis* is set as the reference level (* q<0.05; ** q<0.01; ***q<0.001).

principal ES	HR	95%CI lower	95%CI upper	q	significant
ES-Agat. faecis	1.51	1.12	2.04	3.4×10^{-2}	*
ES-Agat. rectalis	1.18	0.84	1.65	6.0×10^{-1}	
ES-Alis. A putredinis	1.01	0.71	1.44	9.5×10^{-1}	
ES-Bact. H cellulosilyticus	1.45	1.03	2.04	1.2×10^{-1}	
ES-Bact. H stercoris	1.10	0.75	1.60	7.6×10^{-1}	
ES-Bact. H xylanisolvens	0.82	0.53	1.28	6.0×10^{-1}	
ES-Bifi. adolescentis	1.36	0.88	2.10	3.2×10^{-1}	
ES-Blau. A wexlerae	1.32	0.91	1.90	3.2×10^{-1}	
ES-Esch. ruysiae	2.96	2.10	4.18	1.1×10^{-8}	***
ES-Faec. sp000434635	1.35	1.02	1.78	1.2×10^{-1}	
ES-Fime. sp000432435	1.29	0.94	1.76	3.2×10^{-1}	
ES-Flav. plautii	1.31	0.89	1.93	3.2×10^{-1}	
ES-Gemm. A qucibialis	0.93	0.63	1.39	8.2×10^{-1}	
ES-Phoc. A plebeius	1.11	0.78	1.60	7.1×10^{-1}	
ES-Phoc. A vulgatus	1.14	0.81	1.60	6.7×10^{-1}	
ES-Prev. copri	1.62	1.22	2.16	4.9×10^{-3}	**
ES-Prev. rara	0.86	0.56	1.33	6.8×10^{-1}	
ES-Prev. sp003447235	1.83	1.34	2.50	1.5×10^{-3}	**
ES-RUG1. sp900066395	1.05	0.74	1.47	8.4×10^{-1}	

Table S5. Supporting analysis: Cox proportional hazards regression for the continuous univariate signatures (k=20) and genus abundances with respect to total mortality (* q<0.05; ** q<0.01; ***q<0.001). The signals were inverse-rank-transformed to enable comparability between the signatures and genera. Visualized in Figure S2.

name	HR	95%CI lower	95%CI upper	q	significant
ES-Blau. A wexlerae	0.78	0.74	0.83	1.9×10^{-14}	***
ES-Alis. A putredinis	0.85	0.80	0.90	1.5×10^{-7}	***
ES-Agat. rectalis	0.91	0.86	0.96	1.8×10^{-3}	**
ES-Bifi. adolescentis	0.87	0.82	0.92	8.8×10^{-6}	***
ES-Phoc. A vulgatus	0.93	0.88	0.99	1.9×10^{-2}	*
ES-Fime. sp000432435	1.15	1.09	1.22	4.6×10^{-6}	***
ES-Bact. H cellulosilyticus	0.89	0.84	0.95	3.0×10^{-4}	***
ES-RUG1. sp900066395	0.89	0.84	0.94	9.5×10^{-5}	***
ES-Faec. sp000434635	0.99	0.94	1.05	8.6×10^{-1}	
ES-Phoc. A plebeius	0.93	0.87	0.98	1.2×10^{-2}	*
ES-Prev. rara	0.95	0.90	1.00	8.5×10^{-2}	
ES-Esch. ruysiae	1.18	1.11	1.25	2.7×10^{-7}	***
ES-Agat. faecis	0.99	0.94	1.05	8.1×10^{-1}	
ES-Bact. H stercoris	0.97	0.91	1.03	2.9×10^{-1}	
ES-Bact. H xylanisolvens	0.88	0.83	0.93	5.4×10^{-5}	***
ES-Flav. plautii	1.12	1.06	1.18	2.3×10^{-4}	***
ES-Prev. copri	1.14	1.08	1.21	2.4×10^{-5}	***
ES-Gemm. A qucibialis	0.91	0.86	0.97	3.8×10^{-3}	**
ES-Prev. sp003447235	1.25	1.18	1.32	4.4×10^{-13}	***
ES-Bact. H uniformis	0.93	0.88	0.99	2.1×10^{-2}	*
Agathobacter 164117	0.95	0.90	1.01	1.0×10^{-1}	
Alistipes A 871400	0.86	0.81	0.91	9.4×10^{-7}	***
Bacteroides H	0.90	0.85	0.95	7.8×10^{-4}	***
Bifidobacterium 388775	0.88	0.83	0.93	1.7×10^{-5}	***
Blautia A 141781	0.89	0.84	0.94	1.2×10^{-4}	***
Escherichia 710834	1.19	1.12	1.26	4.5×10^{-8}	***
Faecousia	0.93	0.88	0.98	2.0×10^{-2}	*
Fimenecus	1.17	1.10	1.24	7.8×10^{-7}	***
Flavonifractor	0.93	0.88	0.99	2.4×10^{-2}	*
Gemmiger A 73129	0.84	0.79	0.89	3.3×10^{-8}	***
Phocaeicola A 858004	0.93	0.88	0.99	2.4×10^{-2}	*
Prevotella	1.07	1.01	1.13	2.4×10^{-2}	*
RUG115	0.97	0.92	1.03	3.0×10^{-1}	

Table S6. Sensitivity analysis: Cox proportional hazards regression for the continuous multivariate predictors to mortality endpoint using only FINRISK 2002 cohort. FDR corrected p-values are shown; the stars denote the level (* $q<0.05$; ** $q<0.01$; *** $q<0.001$). The test column represents two different settings; using only signatures as predictors (Study year) in the 2002 cohort, and adding the covariates in the model (Covariates). These are separately run for k=5 and k=20. The hazard ratios (HR) for the signatures are reported per 10^6-unit increase in the predictor.

test	name	HR	95%CI lower	95%CI upper	q	significant
Study year	ES-Alis	0.99	0.96	1.02	7.0×10^{-1}	
Study year	ES-Esch	1.04	1.02	1.06	5.2×10^{-4}	***
Study year	ES-Agat	0.97	0.94	1.00	1.3×10^{-1}	
Study year	ES-Prev	1.03	1.01	1.05	7.6×10^{-3}	**
Study year	ES-Bact	1.00	1.00	1.00	4.4×10^{-1}	
Study year	ES-Blau. A wexlerae	0.96	0.93	0.99	3.6×10^{-2}	*
Study year	ES-Alis. A putredinis	0.94	0.91	0.98	1.4×10^{-2}	*
Study year	ES-Agat. rectalis	0.98	0.96	1.01	3.5×10^{-1}	
Study year	ES-Bifi. adolescentis	0.98	0.95	1.01	4.4×10^{-1}	
Study year	ES-Phoc. A vulgatus	1.00	0.96	1.03	8.8×10^{-1}	
Study year	ES-Fime. sp000432435	1.02	1.00	1.04	1.8×10^{-1}	
Study year	ES-Bact. H cellulosilyticus	1.02	1.00	1.04	4.7×10^{-2}	*
Study year	ES-RUG1. sp900066395	0.94	0.91	0.98	1.0×10^{-2}	*
Study year	ES-Faec. sp000434635	1.00	0.98	1.02	8.5×10^{-1}	
Study year	ES-Phoc. A plebeius	1.00	0.98	1.02	9.5×10^{-1}	
Study year	ES-Prev. rara	0.99	0.96	1.01	4.4×10^{-1}	
Study year	ES-Esch. ruysiae	1.04	1.03	1.05	2.3×10^{-8}	***
Study year	ES-Agat. faecis	1.03	1.01	1.06	4.7×10^{-2}	*
Study year	ES-Bact. H stercoris	1.02	1.00	1.04	7.1×10^{-2}	
Study year	ES-Bact. H xylanisolvens	1.00	0.98	1.03	8.5×10^{-1}	
Study year	ES-Flav. plautii	1.01	0.99	1.02	5.2×10^{-1}	
Study year	ES-Prev. copri	1.01	0.99	1.02	5.2×10^{-1}	
Study year	ES-Gemm. A qucibialis	1.00	0.97	1.02	8.1×10^{-1}	
Study year	ES-Prev. sp003447235	1.04	1.03	1.06	1.3×10^{-7}	***
Study year	ES-Bact. H uniformis	1.00	1.00	1.00	9.5×10^{-1}	
Covariates	ES-Alis	0.97	0.93	1.00	1.3×10^{-1}	
Covariates	ES-Esch	1.04	1.02	1.06	3.6×10^{-4}	***
Covariates	ES-Agat	0.99	0.96	1.02	7.3×10^{-1}	
Covariates	ES-Prev	1.01	0.99	1.03	6.8×10^{-1}	
Covariates	ES-Bact	1.00	1.00	1.00	4.4×10^{-1}	
Covariates	Age	1.11	1.11	1.12	1.7×10^{-217}	***
Covariates	Man	1.86	1.66	2.10	1.7×10^{-23}	***
Covariates	BMI	1.02	1.00	1.03	3.6×10^{-2}	*
Covariates	Antibiotics (6 months)	1.11	0.96	1.29	3.5×10^{-1}	
Covariates	ES-Blau. A wexlerae	1.01	0.98	1.03	7.4×10^{-1}	
Covariates	ES-Alis. A putredinis	0.98	0.94	1.02	5.2×10^{-1}	
Covariates	ES-Agat. rectalis	0.97	0.95	1.00	1.2×10^{-1}	
Covariates	ES-Bifi. adolescentis	1.01	0.99	1.03	6.1×10^{-1}	
Covariates	ES-Phoc. A vulgatus	1.01	0.98	1.04	6.8×10^{-1}	
Covariates	ES-Fime. sp000432435	0.99	0.97	1.01	5.2×10^{-1}	
Covariates	ES-Bact. H cellulosilyticus	1.01	0.99	1.02	5.8×10^{-1}	
Covariates	ES-RUG1. sp900066395	0.93	0.90	0.97	1.0×10^{-3}	**
Covariates	ES-Faec. sp000434635	1.00	0.98	1.02	9.5×10^{-1}	
Covariates	ES-Phoc. A plebeius	1.01	0.98	1.03	7.3×10^{-1}	
Covariates	ES-Prev. rara	0.99	0.97	1.01	4.7×10^{-1}	
Covariates	ES-Esch. ruysiae	1.02	1.01	1.04	7.7×10^{-3}	**
Covariates	ES-Agat. faecis	1.01	0.98	1.03	6.8×10^{-1}	
Covariates	ES-Bact. H stercoris	1.02	1.00	1.03	1.1×10^{-1}	
Covariates	ES-Bact. H xylanisolvens	1.02	0.99	1.04	3.0×10^{-1}	
Covariates	ES-Flav. plautii	1.01	1.00	1.02	3.5×10^{-1}	
Covariates	ES-Prev. copri	1.00	0.98	1.01	9.5×10^{-1}	
Covariates	ES-Gemm. A qucibialis	1.00	0.97	1.02	8.1×10^{-1}	
Covariates	ES-Prev. sp003447235	1.04	1.02	1.05	2.8×10^{-5}	***
Covariates	ES-Bact. H uniformis	1.00	1.00	1.00	6.1×10^{-1}	
Covariates	Age	1.11	1.11	1.12	4.5×10^{-217}	***
Covariates	Man	1.85	1.64	2.09	1.6×10^{-22}	***
Covariates	BMI	1.02	1.00	1.03	7.5×10^{-2}	
Covariates	Antibiotics (6 months)	1.11	0.95	1.29	3.5×10^{-1}	
